# General framework of nonlinear factor interactions using bayesian networks for risk analysis applied to road safety and public health

**DOI:** 10.1038/s41598-025-13572-5

**Published:** 2025-08-15

**Authors:** Cinzia Carrodano

**Affiliations:** 1 Geneva School of Economics and Management, University of Geneva, 1205 Geneva, Switzerland; 2 Independent Researcher, 6312 Steinhausen, Switzerland

**Keywords:** Framework, Risk analysis, Nonlinear risk, Bayesian networks, Risk management, Road safety, Applied mathematics, Statistics

## Abstract

In complex systems, understanding the nonlinear interactions among risk factors is essential for accurate risk analysis. However, traditional linear models often fail to capture these complex interdependencies, leading to significant gaps in risk prediction. The aim of this study is to present a novel approach for risk analysis of nonlinear risk interactions using Bayesian networks (BNs), thereby providing a broadly applicable method for risk management and mitigation. Specifically, this study applies a BN-based framework that integrates conditional dependencies and nonlinear effects to illustrate how multifactor risk interactions operate synergistically. Using a step-by-step approach, the interactions among multiple risk factors are first mathematically formalized, and then this framework is applied to a case study of road safety using crash report data. Additionally, a second validation case in public health (type 2 diabetes risk) is included in supplementary materials to illustrate the broader applicability of the framework. The findings demonstrate through BNs and a mathematical framework, how to analyse complex interactions more accurately than traditional methods can, revealing the amplifying or mitigating effects of individual risk factors on outcomes. This approach offers more accurate risk representations and is applicable not only to road safety but also to complex environments, such as healthcare and environmental risk analysis.

**Supplementary Information:**

The online version contains supplementary material available at 10.1038/s41598-025-13572-5.

## Introduction

The goal of risk analysis is to identify, quantify and mitigate potential threats, to minimize their impact on individuals, systems, and societies^1,2^. In complex systems, risk factors often interact in nonlinear ways, producing outcomes that cannot be fully explained by considering various factors in isolation. In the traditional models used by many fields, it is assumed that risk factors act independently and their effects are added. However, in real-world contexts, such as healthcare, environmental science, economics, and road safety, risk factors are rarely independent. Instead, their combined effects significantly deviate from the sum of their individual impacts, suggesting nonlinear interactions. This tendency of traditional analyses to assume independence and linearity among risk factors often leads to underestimations of risk in multifactor situations, leading to inaccurate risk predictions and insufficient mitigation strategies. For example, in road safety, the risks associated with factors such as impaired driving, adverse weather conditions, and lack of safety equipment can interact to amplify the probability and severity of accidents, reflecting a broader trend in which real-world risks are synergistic rather than independent^3^. Other fields exhibit similar complexities, where heterogeneous factors interact in dynamic and nonlinear ways. Such interactions are obvious in healthcare, supply chain management, industrial safety, energy systems, and cybersecurity, as well as in broader domains like pandemics, economics and environmental risks. In these interconnected systems, the combined effect of risk factors often significantly deviates from the sum of their individual impacts. This nonlinearity amplifies risks in ways that cannot be predicted by traditional linear models, where factors are treated in isolation. Over time, these amplified effects can propagate through systems, triggering cascading outcomes, as exemplified by phenomena like the butterfly effect.

Various methodological approaches exist to analyze complex nonlinear interactions between risk factors, including linear models and dynamic simulation models^4^. Recent research in risk and reliability analysis include the application of machine learning-based approaches such as graph neural networks^5^ who developed a GNN surrogate model to perform seismic analysis of highway bridge systems. He and Weng^6^ proposed a model for multi-hazard coupling disasters using a Choquet integral regression method, which addresses the problem of nonlinear additivity of risks by considering magnification effects on disaster severity and victim vulnerability. Although these approaches are highly effective, Bayesian networks (BNs) provide distinct advantages such as probabilistic reasoning, graphical representation of causal relationships, and precise quantification of conditional probabilities. Given these strengths, the current study focuses specifically on the rigorous mathematical formalization of nonlinear interactions using BN.

Previous research explicitly highlighted the difficulties inherent in assessing multivariate and multi-hazard risks, such as Kappes et al*.*^7^, who emphasize the need for integrated and harmonized methodological approaches. The proposed general framework aims to provide a clear advancement in multi-factor risk assessment methodologies.

Traditional risk assessment models and analysis often consider risk factors in isolation, leading to underestimations of the true risk and inadequate prevention strategies^8,9^. This highlights the limitations of linear approaches and underline the critical need for a unified framework capable of analyzing nonlinear interactions across domains, enabling more accurate risk assessment and effective mitigation strategies. Thus, capturing these nonlinear interactions is crucial for accurate risk assessment and analysis, as well as the development of effective intervention strategies in various complex scenarios.

One promising approach for modelling the nonlinear interactions between factors is BNs. BNs can effectively address this issue, as they inherently account for complex interdependencies, enabling more precise representations of how risk factors interact and influence other factors in multifactor scenarios. Owing to their ability to model intricate dependencies among variables, BNs are robust frameworks that incorporate conditional dependencies, making them well suited for analysing multifactor risks in complex systems^10–12^. Unlike linear models, BNs can incorporate both conditional dependencies and nonlinear effects, offering a more realistic view of how risk factors operate in tandem to produce adverse outcomes.

Notably, Bayesian methods have been increasingly applied to capture the complex, nonlinear relationships among road safety risk factors. For example, Bayesian hierarchical models, spatiotemporal models, and random parameter models have been utilized to explore complex dependencies, space‒time interactions, and regional variations in crash frequencies^13–16^.

In road safety, BNs capture interactions among driver behavior, environmental conditions, and technical specifications to predict accident severity and inform targeted interventions^17–19^. They have also been widely utilized to evaluate transportation risks, including hazardous material transportation, marine, and railway safety^20–24^. Previous research^3,25,26^ has specifically advanced road safety analyses through BNs, demonstrating the capability of these networks to model nonlinear relationships between risk factors. Similarly, BNs have been increasingly applied in environmental science, demonstrating their ability to integrate various knowledge types^27^, and address complex ecological and socioeconomic risks^28^ or modeling climate change impacts^29^. In healthcare, BNs have elucidated interactions between genetic, and environmental factors, enhancing personalized medicine and understanding synergistic health outcomes^30–32^. Additionally, BNs have shown utility in assessing risks in various fields such as process industry safety^33^, supply chain risk^34^, high-stakes infrastructure risk^35^, steel construction safety^36^, and nuclear waste risk^37^. These wide-ranging applications underscore the adaptability and robustness of BNs, highlighting their potential as a unified methodological framework for analyzing complex nonlinear risk interactions across many domains.

However, despite significant advances in risk assessment, a unified framework that formalizes a data-driven nonlinear risk interactions analysis across different domains has not yet been developed. In this work, we aim to advance the current understanding of risk by presenting a novel general framework of Nonlinear Factor interactions based on BNs for Risk analysis (NFBR). Extending the previous work^3^, where we demonstrated the nonlinearity of risk interactions in road safety, we present a broader data-driven framework for modelling nonlinear risk interactions across various domains. While BNs generally offer robust probabilistic frameworks, this work develops a mathematical framework that captures more complex interactions among multiple risk factors, providing a comprehensive tool for risk analysis across several fields based on BNs. In the work^3^, we have demonstrated how multiple interacting risk factors can produce nonlinear outcomes, amplifying or attenuating the overall risk. This general framework provides a unified foundation for understanding complex risk dynamics, offering a more precise approach to risk analysis and mitigation. To validate the performance of the proposed framework, we apply the general framework to road safety analysis, leveraging crash report data from the state of Maryland. This case study, which was originally used in the previous paper^3^, is ideal for testing the general framework, as road safety risks are inherently multifaceted, encompassing human behaviour, environmental conditions, and vehicle factors. The results elucidate interactions among risk factors, revealing both the nonlinear nature of these interactions and potential interventions for mitigating adverse outcomes. While BNs have been applied in risk assessment, this work introduces an added mathematical extension that formalizes nonlinear interactions among risk factors, improving the precision and generalizability of complex risk analysis, which may considerably advance risk analysis and management in various scenarios.

While road safety serves as the primary demonstration in this paper, the general framework is designed for broad application across contexts involving multiple risk factors. For example, this framework can be applied to healthcare risk evaluation, as biological, environmental exposure, and lifestyle factors interact in complex ways to influence disease onset and progression or pandemics risks. This framework can also be applied in many other complex environments and domains, such as finance, cybersecurity, energy systems, or urban planning are further examples where interacting factors produce nonlinear risks, making a comprehensive, cross-domain framework essential for the development of effective risk management strategies, as well as the previously mentioned examples.

The remainder of this paper is structured as follows. First, we outline the methodology used to develop a general framework that captures nonlinear interactions among multiple risk factors, structured in a step-by-step approach. The first step, we generalize the mathematical demonstration from the previous paper^3^. Next, we introduce a nonlinear effect factor to enhance the model, focusing on interactions between two risk factors. Finally, we expand this framework to accommodate multiple interacting factors, establishing the complete mathematical foundation. In the Results section, we apply this framework to road safety analysis, examining three scenarios and revealing the complex dynamics among interacting risk factors, followed by a detailed analysis. The paper concludes with a discussion of the broader implications of the findings for risk assessment and management across several domains.

## Methods

### Introduction and the case study applied to the general framework development

This section presents the methodology used to develop the general framework of nonlinear risk interactions based on BNs. The goals of this approach are to formalize the nonlinear nature of interactions among multiple risk factors and to provide a framework applicable to various fields. The mathematical framework builds on traditional BN structures by introducing interaction terms that capture nonlinear dependencies among risk factors. These terms enable the model to quantify how combined risk factors produce amplified or mitigated effects, refining the predictive accuracy of BNs in complex scenarios.

The dataset used in this study is the same as the one in the previous research^3^, which focused on exploring nonlinear risk factor interactions in road safety using BNs. This dataset consists of 26,746 crash reports collected in Maryland during the first quarter of 2018 from the *data.gov* website. It includes detailed information on driver behavior, environmental conditions, vehicle characteristics, and crash outcomes. The key variables are driver age, gender, physical condition, road geometry, surface conditions, weather, light conditions, and vehicle type, safety equipment use, and vehicle movement. The target variables are the collision types and severity of crashes. For more detailed information about the dataset, please refer to the previous research^3^. In the prior study, this dataset allowed us to identify nonlinear relationships among risk factors, such as the amplified risk of accidents due to interactions between driver fatigue and snowy conditions. However, the analysis was limited to domain-specific scenarios. The current research builds upon these findings, aiming to generalize the methodology for broader applications across various domains of risk analysis.

### Framework development

**Fig. 1:**
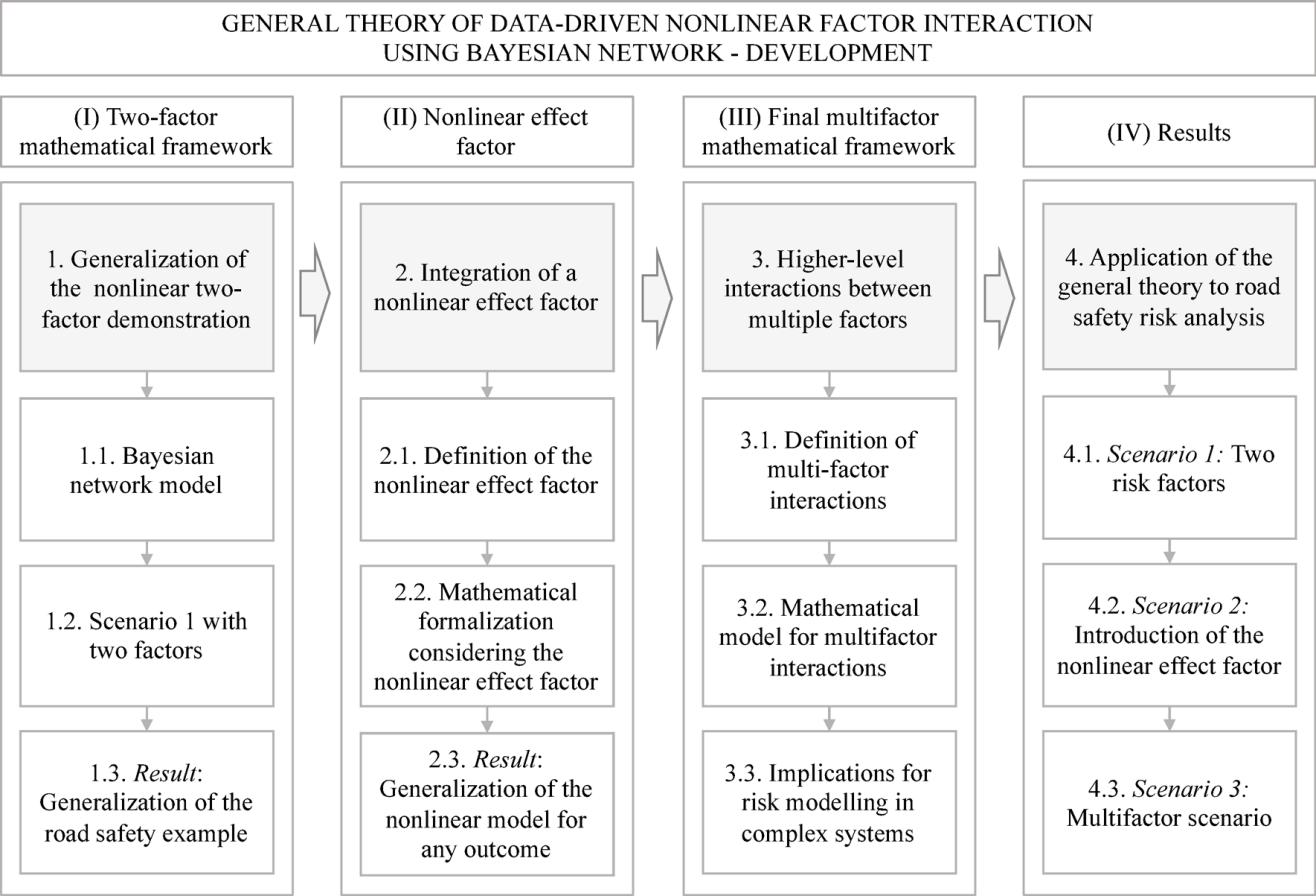
Methodology for the development of the general framework in four phases. Phases I to III are detailed in Section "Results" and phase IV is detailed in Section "Discussion and conclusion".

The development of the proposed general framework involved four phases: (I) the development of a two-factor mathematical framework to understand the nonlinear nature of interactions between two risk factors; (II) the integration of a nonlinear effect factor in the previous model; (III) the integration of higher-level interactions among multiple risk factors in the final mathematical model; and (IV) the application of the final model in a real-world scenario (Fig. 1). By using BNs, this approach captures both the probabilistic dependencies between factors and their nonlinear interactions.

These phases are detailed below. Phases I to III are explained in Section "Results" hereafter, and the phase IV is explained in Section "Discussion and conclusion".

#### Phase I: generalization of the two-factor nonlinear risk evaluation method

In the first step, the nonlinear nature of risk is demonstrated using two interacting factors on the basis of the method presented in^3^. First, the BN model is described, and then the mathematical framework considering two risk factors is presented.

#### Phase II: introduction of a nonlinear effect factor

In the second step, the concept of a nonlinear effect factor is introduced. This factor quantifies the extent to which the combined effect of the interacting risk factors differs from their linear, additive combination. The nonlinear effect factor is defined and integrated into the mathematical model for road safety. A generalization of the nonlinear model for any scenario is then presented.

#### Phase III: higher-level interactions between multiple factors

In the third step, the mathematical framework is extended to include interactions between more than two risk factors, allowing for a more comprehensive view of complex risk scenarios. Multifactor interactions are defined, mathematical models are developed, and the implications for risk modelling are discussed.

#### Phase IV: application of the general framework in real-world scenarios

Finally, the general framework is applied to a real-world scenario, specifically road safety, to demonstrate its applicability in practical settings. The same road safety example as presented in the previous paper^3^ is used. Three scenarios are considered based on the above three steps in the general framework: scenario 1, which involves two risk factors; scenario 2, which involves the incorporation of the nonlinear effect factor; and scenario 3, which involves the development of a multifactor risk interaction model.

This methodology forms the foundation of the general framework of nonlinear risk interactions using BNs. The analysis demonstrates how BNs can effectively model the complex dynamics of multiple interacting risk factors across various domains.

## General framework of data-driven nonlinear factor interactions using Bayesian networks

### Phase I: generalization of the nonlinear two-factor demonstration (based on^3^)

In this phase, the concept of nonlinear risk is generalized using BNs, following the foundational example presented in^3^. This framework moves beyond traditional linear models to capture the complex interactions among multiple risk factors. First, the BN model is established; then, nonlinear computations are applied to illustrate how risk factors interact in a nonadditive manner.

#### Bayesian network model

A BN is a probabilistic graphical model that represents a set of variables (risk factors) and their conditional dependencies via a directed acyclic graph (DAG). Each node in the network represents a risk factor, while the edges represent the probabilistic dependencies between the factors.

For a given set of variables $X=\left\{{X}_{1},{X}_{2},{\dots ,X}_{n}\right\}$, the joint probability distribution is expressed as:

1 $$P(X)=\prod_{i=1}^{n}P\left({X}_{i}|parents({X}_{i})\right)$$

where $P\left({X}_{i}|parents({X}_{i})\right)$ is the conditional probability of factor *X*_*i*_ given its parent nodes.

This structure captures the causal dependencies between risk factors, with the assumption that each variable is conditionally independent of its non-descendants given its parent nodes^33^. The BN allows us to model these dependencies and compute the combined probability of outcomes on the basis of multiple interacting risk factors.

#### Scenario 1: demonstration of nonlinear nature of risk using two risk factors (based on^3^)

When the combined effect of two or more risk factors is different from the sum of their individual effects, there is a nonlinear relationship between those factors. To demonstrate this nonlinearity, the road safety example from^3^ is used, considering two key risk factors:

- *X*_*1*_ is a human risk factor, such as driving under the influence (DUI);
- *X*_*2*_ is an environmental risk factor related to road conditions, such as snow.

In a traditional linear model, the combined effect of these two factors is additive. Equation (2) quantifies the additive scenario as the sum of the individual increases in risk (variation in probability compared with a normal situation) associated with each risk factor independently. This ensures a rigorous definition of the additive baseline against which nonlinear interactions are measured:

2 $$\Delta P\left(accident|{X}_{1}, {X}_{2}\right)=\Delta P\left(accident|{X}_{1}\right)+\Delta P\left(accident|{X}_{2}\right)$$

where $\Delta P\left(accident|{X}_{1}\right)$ and $\Delta P\left(accident|{X}_{2}\right)$ represent the increases in the probability of an accident due to each risk factor separately, relative to the baseline (normal situation, e.g., normal human state and dry road conditions).

However, the real-world interactions between these factors are often nonlinear. In a nonlinear model, their combined effect can deviate from the sum of their individual effects:

3 $$\Delta P\left(accident|{X}_{1}, {X}_{2}\right)\ne \Delta P\left(accident|{X}_{1}\right)+\Delta P\left(accident|{X}_{2}\right)$$

where $\Delta P$ represents the difference in the probability of an accident due to the presence of a risk factor compared with the risk in a normal situation (i.e., normal human state and dry road conditions). This reflects the nonlinear nature of risk, as considering the interaction between *X*_*1*_ and *X*_*2*_ results in a total risk that is greater or less than the simple sum of their independent effects.

The example in^3^ is related to road safety and, more specifically, the combined effect of human risk factors (e.g., normal state, fatigued state, or DUI) and environmental risk factors (e.g., road conditions such as dry or snowy). In this case, DUI combined with snowy road conditions results in a disproportionately high risk of accidents, with a much higher risk than would be predicted by summing their individual effects.

### Phase II: integration of a nonlinear effect factor in the mathematical model with two risk factors

In this phase, a nonlinear effect factor $(\gamma )$ is introduced. This factor represents a methodological innovation for capturing and understanding nonlinear interactions among risk factors. This factor allows for a clear differentiation between additive and non-additive scenarios, highlighting how interactions amplify or attenuate overall risk in complex systems.

#### Definition of the nonlinear effect factor

The nonlinear effect factor $(\gamma )$ quantifies the deviation from a linear combination of risk factors by comparing the combined effect of multiple factors to their additive effect relative to the normal situation. The nonlinear effect factor is mathematically defined as:

4 $$\gamma =\frac{\Delta (Combined effect)}{\Delta (Additive effect)}$$

where $\Delta (Combined effect)$ represents the difference in risk between the combined scenario (e.g., two or more interacting risk factors) and the linear additive scenario, thus precisely quantifying the deviation from linearity. The $\Delta \left(Additive effect\right)$ represents the sum of the individual effects of the risk factors, assuming that there is no interaction between them (i.e., a linear model).

In particular, the proposed mathematical framework explicitly accommodates cases of pure synergy, in which the additive effect $\Delta \left(Additive effect\right)$ is zero, but the combined effect $\Delta \left(Combined effect\right)$ is non-zero. Such scenarios occur when individual risk factors do not independently increase risk significantly, yet their interaction results in an increase in risk. Capturing this form of pure synergistic interaction explicitly is an essential feature of the nonlinear effect factor $\gamma$. Mathematically, in these cases, $\gamma$ tends toward infinity. Practically, these scenarios are interpreted as purely synergistic interactions, highlighting that risk emerges exclusively from the interaction itself, with no finite numerical value of $\gamma$ being provided.

This factor determines whether the interaction between risk factors amplifies or attenuates the overall risk. In particular, $\gamma >1$ indicates that the risk is amplified, meaning that the combined effect is greater than the sum of the individual effects; $\gamma <1$ indicates that the risk is attenuated, meaning that the combined effect is less than the sum of the individual effects; and $\gamma =1$ indicates a linear interaction, meaning that the combined effect is equal to the sum of the individual effects.

This *delta-based* approach provides a formal framework for quantifying by how much the combined effect of the risk factors deviates from their additive interaction, capturing the nonlinear nature of risk.

#### Mathematical formalization considering the nonlinear effect factor

To model a nonlinear scenario, the nonlinear effect factor $\gamma$ is incorporated into the additive model to account for the nonlinear amplification or attenuation of the risk in the combined case. The combined effect of two risk factors *X*_*1*_ and *X*_*2*_ is computed as:

5 $$\Delta P\left(accident|{X}_{1}, {X}_{2}\right)=\gamma \cdot (\Delta P\left(accident|{X}_{1}\right)+\Delta P\left(accident|{X}_{2}\right))$$

where $\Delta P\left(accident|{X}_{1}, {X}_{2}\right)$ represents the variation in the probability of an accident when both *X*_*1*_ and *X*_*2*_ are present; $\Delta P\left(accident|{X}_{1}\right) \text{and} \Delta P\left(accident|{X}_{2}\right)$ are the variations in the probability when each factor is considered individually; and $\gamma$ is the nonlinear effect factor that modulates the interactions between the two factors.

Although Eq. (5) structurally resembles a linear combination, it incorporates the nonlinear effect factor $(\gamma$). This factor captures deviations from pure linearity and thus directly models the intensity of nonlinear interactions among risk factors, making explicit how the combined effect differs from a simple sum of individual effects.

#### Generalization of the nonlinear model for any outcome

To extend the applicability of the nonlinear interaction model beyond the specific example of road accidents, the model is generalized to apply to any outcome, including health conditions, environmental impacts, and other complex risk-related events.

While the example from^3^ focuses on road safety, this mathematical framework can be applied to any domain involving interactions among multiple risk factors. For example, in healthcare, the interaction between pre-existing medical conditions and environmental factors (e.g., pollution or lifestyle choices) may result in nonlinearly increased risks of adverse health outcomes.

Similarly, in environmental management, the interaction between climate change-related factors and deforestation or industrial pollution can increase the risk of ecosystem degradation. To formalize these interactions, a nonlinear effect factor is introduced, which quantifies the extent to which the combined effect of multiple risk factors deviates from the additive model.

The general equation for the outcome’s risk, $P(outcome)$, is a function of the two interacting risk factors *X*_*1*_ and *X*_*2*_ and is defined as:

6 $$\Delta P\left(outcome|{X}_{1}, {X}_{2}\right)=\gamma \cdot (\Delta P\left(outcome|{X}_{1}\right)+\Delta P\left(outcome|{X}_{2}\right))$$

where $\Delta P\left(outcome|{X}_{1}, {X}_{2}\right)$ represents the variation in the probability of the outcome when both *X*_*1*_ and *X*_*2*_ are present; $\Delta P\left(outcome|{X}_{1}\right)+\Delta P\left(outcome|{X}_{2}\right)$ represents the individual contributions of the respective factors to the probability of the outcome; and $\gamma$ is the nonlinear effect factor that quantifies the deviation from the additive model.

In this formulation, $\gamma >1$ indicates that the combined effect of *X*_*1*_ and *X*_*2*_ results in a greater probability of the outcome than that predicted by the additive model, indicating risk amplification; $\gamma <1$ indicates that the combined effect of *X*_*1*_ and *X*_*2*_ results in a lower probability of the outcome than that predicted by the additive model, indicating risk attenuation; and $\gamma =1$ indicates that the interaction between the two factors is linear, meaning that the combined effect is exactly the sum of the individual effects.

This generalized nonlinear model provides a framework applicable to any scenario, allowing the model to be used in various fields, such as healthcare (e.g., risk of disease) and environmental management (e.g., risk of ecosystem failure).

### Phase III: higher-level interactions between multiple factors (multifactor model)

In this phase, the framework of nonlinear risk interactions is extended, and a generalized model is introduced, considering the nonlinear relationships among multiple interacting risk factors *X*_*1*_*, X*_*2*_*, X*_*3*_*,…,X*_*n*_​. The same principle is applied in this step; namely, the combined effect of multiple factors is not simply additive but instead follows a nonlinear relationship, with the interactions among factors amplifying or modulating the overall outcome.

#### Definitions of multifactor interactions

Multifactor interactions occur when several factors simultaneously influence an outcome in a nonlinear manner. Rather than each factor contributing independently to the outcome, the presence of other factors can either amplify or attenuate their combined effects. For example, in the context of health risk modelling, factors such as smoking, physical inactivity, and genetic predisposition interact to nonlinearly increase the risk of heart disease. The effects of these factors are not simply added; instead, they result in a synergistic effect that is often greater than the sum of the individual risks. This concept is particularly relevant in complex systems, as multiple interacting variables drive the overall behaviour of the system. In healthcare, environmental safety, and transportation, the interactions among multiple factors are often nonlinear, and understanding these relationships is critical for making accurate predictions and developing effective interventions.

#### Mathematical model considering multifactor interactions

The combined probability of a critical event (or outcome) considering *n* interacting risk factors can be expressed as:

7 $$P\left(outcome\right)=f\left({X}_{1},{X}_{2},\dots ,{X}_{n}\right)$$

where $P\left(outcome\right)$ represents the probability of the outcome (e.g., an adverse event such as an accident, disease, or environmental hazard) and $f\left({X}_{1},{X}_{2},\dots ,{X}_{n}\right)$ is a function that represents the interactions among the *n* risk factors.

The mathematical formulation for modelling the variation in the probability of the outcome considering multiple interacting factors is given by:

8 $$\Delta P\left(outcome|{X}_{1},{X}_{2},\dots ,{X}_{n}\right)=\gamma \cdot \sum_{i=1}^{n}\Delta P\left(outcome|{X}_{i}\right)$$

where $\Delta P\left(outcome|{X}_{1},{X}_{2},\dots ,{X}_{n}\right)$ represents the variation in the probability of the outcome when all *n* factors are present compared with the probability in the normal situation; $\sum_{i=1}^{n}\Delta P\left(outcome|{X}_{i}\right)$ is the sum of the individual effects of each factor ${X}_{i}$, assuming no multifactor interactions (i.e., a linear model); and $\gamma$ is the nonlinear effect factor, which modulates the overall combined effect of the risk factors and captures the amplification or attenuation of the risk due to their interactions.

This formulation shows that the presence of multiple interacting factors results in an outcome that can significantly deviate from the simple sum of the individual probabilities. The interactions among the factors introduce nonlinear effects, with outcomes that may be much greater or much smaller than what would be predicted by a purely additive model.

#### Implications for risk modelling in complex systems

The general framework of nonlinear risk based on BNs provides a comprehensive framework for understanding risk behaviour in complex systems. This approach allows for the modelling of both the nonlinearity in factor interactions and the conditional dependencies inherent in real-world systems. By capturing the interactions among multiple factors, this framework can be applied across a variety of contexts. For example, in healthcare, this model can be applied to represent the interplay among genetic predispositions, lifestyle choices, and environmental exposure factors to predict disease risk. In the context of road safety, this approach can help understand how human behaviour, environmental conditions, and vehicle safety features interact to increase the likelihood of accidents. Moreover, in the context of environmental risk, this method can be used to evaluate how factors such as climate change, deforestation, and industrial pollution interact to increase threats to ecosystems. By providing a probabilistic representation of these complex interactions, the proposed BN-based model offers a powerful tool for predicting outcomes and guiding interventions in scenarios with interactions among multiple risk factors. This general framework serves as a foundation for future research and application in various domains, offering insights into the nonlinear dynamics that govern complex systems.

## Results

In this section, the general framework of nonlinear risk using BNs is applied to demonstrate nonlinear interactions among risk factors. The steps described correspond to the framework developed in the previous sections and the Phase IV of the methodology. Specifically, this case study of road safety^3^ serves as an initial validation of the enhanced BN framework. This example illustrates how nonlinearly interacting risk factors affect the likelihood of accidents. By applying real-world data, the model’s ability to capture complex risk interactions is demonstrated, setting a foundation for similar applications in healthcare, environmental science, and other fields. The modelling begins with two-factor interactions and then progressively introduces the concept of multifactor risk interactions.

### Step I: nonlinear risk model with two risk factors – DUI and snowy road conditions

To demonstrate the core principles of the general framework, the interaction between two key risk factors is first modelled with a BN. This first scenario replicates the scenario presented in^3^. The two factors in this scenario are the driver’s physical condition (normal or DUI) and the road surface condition (dry or snowy).

**Fig. 2:**
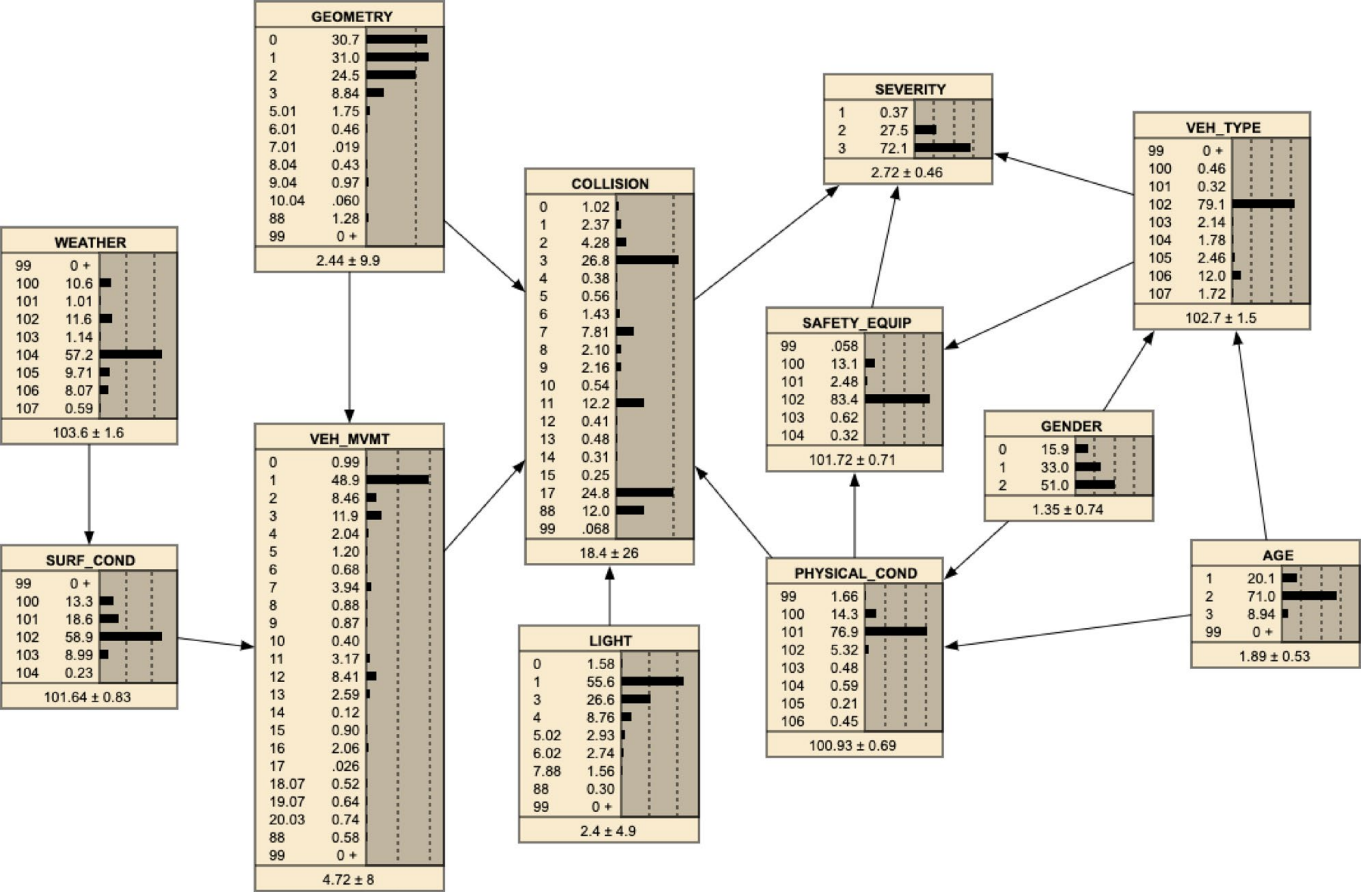
Bayesian network model, as originally published in^3^. This BN is reproduced here as a visual reference, facilitating understanding of the novel mathematical formalization and rigorous quantification of nonlinear interactions introduced in this manuscript: the calculation of the nonlinear effect factor and its generalization to multifactor interactions.

In the BN model for this scenario, each node represents a risk factor (e.g., the driver’s state or road surface condition), and the edges represent the probabilistic dependencies between these factors (Fig. 2). This framework enables us to model how these risk factors influence each other and contribute to the overall probability of an accident.

#### Interactions between two risk factors

In this scenario, the nonlinear nature of risk is analysed when two factors, DUI and snowy road conditions, are both present. The probability of an accident is modelled on the basis of the following conditions:

- DUI only: A driver’s impaired state increases the risk of an accident under normal (dry) road conditions.
- Snowy road conditions only: This environmental factor increases the risk of an accident when the driver is in a normal physical state.
- Both DUI and snowy road conditions: The combined effect of the driver’s impaired state and snowy road conditions leads to a nonlinear change in accident risk, which differs from the simple sum of the individual effects of these factors.

The combined effect of DUI and snowy conditions results in a much greater probability of an accident than that predicted by the additive model (i.e., if the factors were independently added). This clearly demonstrates the nonlinear amplification of risk when interactions among two factors are considered.

#### Bayesian network representation

The BN model, as shown in Fig. 2, illustrates the probabilistic dependencies between various risk factors, including physical conditions, surface conditions, safety equipment, weather conditions, and other contributing variables. Each node represents a specific variable, while the edges indicate the probabilistic dependencies between them. This network allows us to compute the joint probability distribution across all factors, enabling a comprehensive understanding of how these factors interact.

#### Scenario 1: nonlinear interaction between DUI and snowy conditions

Using the BN model, the combined effect of DUI and snowy road conditions on accident severity was evaluated. The results are presented in Table 1, which displays the probabilities of different outcomes (e.g., severity: fatal accidents, injuries, and damage-only accidents) considering various risk factors.

**Table 1:**
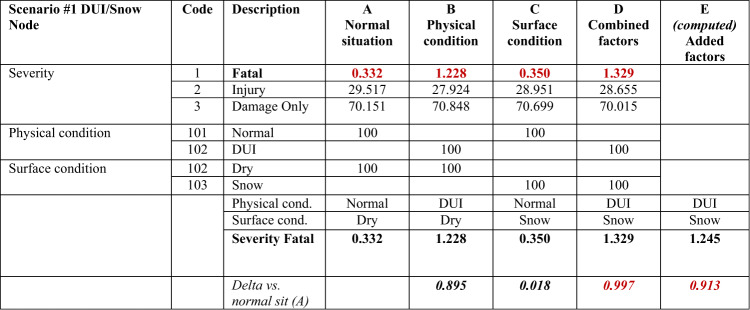
Nonlinear risk factor results for Scenario #1 (DUI/Snow), as previously published in^3^.

Significant values are in bold and italic.

The columns in Table 1 show the following:

- Column A: Probabilities of fatal accidents under normal conditions (driver in a normal state and dry road conditions).
- Column B: Probabilities of fatal accidents under the DUI and dry road conditions.
- Column C: Probabilities of fatal accidents under snowy road conditions when the driver is in a normal physical state.
- Column D: The combined effect of DUI and snowy road conditions on the probability of fatal accidents.
- Column E: The additive effect of both conditions individually on the probability of fatal accidents.

In this example, the variation of the combined probability of an accident is calculated using Eq. (3) as illustrated in Table 1:

9 $$\Delta P\left(accident|{X}_{1}, {X}_{2}\right)\ne \Delta P\left(accident|{X}_{1}\right)+\Delta P\left(accident|{X}_{2}\right)=0.997\ne (0895+0.018)$$

where *X*_*1*_ is the physical state risk factor (DUI), *X*_*2*_ is the road condition (snowy), and $P\left(accident|{X}_{1}, {X}_{2}\right)$ is the variation in the accident probability due to risk factors *X*_*1*_ and *X*_*2*_.

The variation of the probability obtained from adding the effects of each factor compared to the probability in the normal situation is 0.913. However, the variation of the actual relative probability considering when both factors are present is 0.997 (Table 1). The difference between these two results demonstrates the nonlinearity of the interaction between these factors, namely, that the combined effect is not simply the sum of the effects of the individual risk factors, confirming the nonlinear nature of risk.

For further details on the BN model and its results, refer to the original paper^3^.

### Step II: introduction of the nonlinear effect factor

In this step, the previous mathematical model is extended by introducing the nonlinear effect factor (γ), which quantifies the extent to which the combined effect of the interacting risk factors exceeds the additive effect of these factors. The calculation process for the nonlinear effect factor is first explained on the basis of Eq. (4); then, a mathematical modelling application is presented.

#### Nonlinear effect factor calculation with the delta-based method

To assess the nonlinear interactions between risk factors, the nonlinear effect factor (γ) is calculated by comparing the combined effect of the risk factors to the sum of their individual effects (i.e., the additive model). This novel approach is applied to a road safety example, and the calculated values for the combined effect and additive effect are 0.997 and 0.913, respectively (Table 1). Thus, γ can be calculated using Eq. (4), as follows:

10 $$\gamma =\frac{\Delta (Combined effect)}{\Delta (Additive effect)}=\frac{0.997}{0.913}=1.092$$

This result shows that the combined effect is 9.2% greater than the sum of the individual effects (additive model). Therefore, the nonlinear effect factor of 1.092 indicates that the interaction between the driver state (DUI) and road condition (snowy) increases the overall risk by 9.2% compared with the risk predicted by simply adding their individual effects.

#### Mathematical model with the nonlinear effect factor applied to the road safety example

When applying the nonlinear effect factor of 1.092 in the road safety example, Eq. (5) yields:

11 $$\Delta P\left(accident|{X}_{1}, {X}_{2}\right)=\gamma \cdot \left(\Delta P\left(accident|{X}_{1}\right)+\Delta P\left(accident|{X}_{2}\right)\right) =1.092 \cdot \left(0.895+0.018\right)=0.997$$

This confirms that the combined effect of the driver in a DUI state and snowy road conditions on the probability of an accident is 9.2% greater than the sum of their individual effects, validating the nonlinear amplification of risk in this scenario.

This finding highlights the importance of incorporating nonlinear effect factors into risk models, as they provide a more accurate representation of how interacting risk factors amplify overall risk.

### Step III: multifactor road accident scenario

In this step, a multifactor scenario is modelled to demonstrate how more than two risk factors, namely, a DUI driver state, snowy road conditions and the absence of a seatbelt (no belt), interact nonlinearly to increase the severity of accidents. In contrast to traditional models, in which it is assumed that the overall risk is the sum of the risk of each individual factor, the proposed approach considers how the effects of each factor amplify the effects of the other factors, leading to greater risk than that predicted by the additive model.

#### Risk factors and evidence variables

In this example, the risk factors can be categorized as follows:

- Human factor: DUI.
- Environmental factor: Snowy road condition.
- Safety equipment factor: No seatbelt use.

These three factors interact to increase the probability of fatal accidents. The results, which are presented in Table 2, are obtained based on specific evidence variables (set at 100% in the table), demonstrating the interactions among these factors.

**Table 2:**
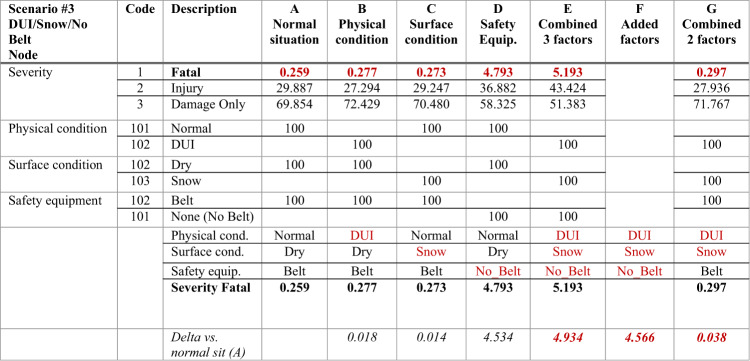
Nonlinear risk factor results for Scenario #3 (DUI/Snow/No Belt) considering multifactor interactions.

Significant values are in bold and italic.

#### Multifactor interaction results

The results of the multifactor interaction are summarized in Table 2, which provides a detailed comparison of individual and combined effects for each risk factor. The table below breaks down the contributions of the driver being in the DUI state, snowy road conditions, and the absence of a seatbelt, both individually and collectively, allowing us to observe the nonlinear amplification of risk.

Table 2 presents the results for Scenario #3, where the columns are described as follows:

- Column A: Represents the normal situation, where none of the risk factors are present (normal physical condition, dry road and the driver wearing a seatbelt).
- Columns B, C, D: Show the individual effects of each risk factor. Column B reflects DUI only, column C shows the snowy condition only, and column D reflects the absence of a seatbelt.
- Column E: Demonstrates the combined effect when all three risk factors (DUI, snow and no seatbelt) are all present.
- Column F: Represents the added effects of all factors considered individually for comparison.
- Column G: Shows the scenario in which DUI and snowy conditions are present but a seatbelt is used.

#### Nonlinear amplification of risk

To further explore the nonlinear amplification of risk, this analysis aimed to quantify the difference between the effects of interacting risk factors (nonlinear model) and their combined effect factors (additive model). Highlighting these differences demonstrates the significance of incorporating nonlinear interactions in risk analysis models.

The table below (Table 3) highlights the nonlinear nature of risk by comparing the added factor effect (Table 2, Column F) and the combined factor effect (Table 2, Column E). The difference between these two factors demonstrates how much higher the risk is when the factors interact nonlinearly (BN model) than when they are treated as independent (additive model).

**Table 3:** Nonlinear effect factor calculation for 2 and 3 risk factors.

	Severity fatal		3 Factors variation		2 Factors variation
Fatalities in normal situations	0.259		–		–
Fatalities with DUI	0.277	*DUI*	0.018	*DUI*	0.018
Fatalities with snowy road condition	0.273	*Snow*	0.014	*Snow*	0.014
Fatalities with no seatbelt	4.793	*No Belt*	4.534	*Belt*	
Added factor effect (a)			4.566		0.032
Combined factor effect (b)	5.193		4.934		0.038
**Difference (a) – (b)**			**− 0.368**		**− 0.006**
Nonlinear effect factor *(delta-based method)*					
Delta combined factor effect			4.934		0.038
Delta added factor effect			4.566		0.032
*Delta combined factor effect / delta added factor effect*			***1.081***		***1.187***
***Percentages***			***8.10%***		***18.70%***

Significant values are in bold and italic.

This comparison captures the nonlinear risk effect, with the combined risk effect being different (higher) than the added risk effect in the case of both three risk factors and two risk factors.

#### Nonlinear effect factor calculation

To quantify the degree of nonlinearity, the nonlinear effect factor is calculated using the *delta-based* method for the three-factor scenario based on Eq. (4):

12 $$\gamma =\frac{\Delta (Combined effect)}{\Delta (Additive effect)}=\frac{4.934}{4.566}=1.081$$

Considering the three factors (DUI, snow and no seatbelt), the values can be substituted into the generalized Eq. (8):

13 $$\begin{aligned} \Delta P\left( {accident|X_{1} ,X_{2} ,X_{3} } \right) & = \gamma \cdot \left( {\Delta P\left( {accident|X_{1} } \right) + \Delta P\left( {accident|X_{2} } \right) + \Delta P\left( {accident|X_{3} } \right)} \right) \\ & = 1.081 \cdot \left( {0.018 + 0.014 + 4.534} \right) = 4.934 \\ \end{aligned}$$

where *X*_*1*_ is the human factor (DUI), *X*_*2*_ is the environmental factor (snowy road condition), *X*_*3*_ is the safety equipment factor (no seatbelt), and 1.081 represents the nonlinear effect factor ($\gamma$), which was previously calculated.

For the two-factor scenario (DUI and snow) with a seatbelt, the calculation is based on Eq. (5):

14 $$\begin{aligned} \Delta P\left( {accident|X_{1} ,X_{2} } \right) & = \gamma \cdot \left( {\Delta P\left( {accident|X_{1} } \right) + \Delta P\left( {accident|X_{2} } \right)} \right) \\ & = 1.187 \cdot \left( {0.018 + 0.014} \right) = 0.038 \\ \end{aligned}$$

These calculations underscore the significant impact of nonlinear interactions among multiple risk factors, particularly in a real-world scenario such as road safety. The broader implications of these findings are analysed next, and the discussion addresses how this nonlinear amplification of risk can be leveraged to achieve more accurate risk assessments and develop effective intervention strategies.

#### Analysis of the multifactor results

The results of the case involving the DUI driver state, snowy road conditions, and absence of a seatbelt elucidate the complex dynamics of risk in real-world road safety scenarios. Traditional linear models often fail to capture the synergistic effects that arise when multiple risk factors cooccur. The findings confirm that the interactions among multiple risk factors produce outcomes that considerably differ from those predicted by simple additive models, underscoring the nonlinear nature of real-world risk.

The most striking observation from this multifactor scenario is the nonlinear amplification of risk when all three factors (DUI, snowy road conditions and the absence of a seatbelt) are present simultaneously. The probability of a fatal accident in this scenario predicted by the combined effects model increases to 5.193, compared with a baseline probability of 0.259 under normal conditions (normal physical conditions, dry road conditions and the use of a seatbelt). The difference in probability between the normal situation and the probability predicted by the additive model for these three factors is 4.566. However, the difference in probability between the normal situation and that predicted by the combined risk model reaches 4.934; specifically, there is an 8.1% increase in the risk of a fatal accident when considering nonlinear interactions compared with the risk predicted by the additive model. This amplification becomes particularly important when the role of each factor is considered. The DUI and snowy conditions have already been established as significant contributors to accident risk. However, when these factors are combined with the absence of a seatbelt, the probability of an accident increases dramatically rather than being merely added.

Furthermore, the absence of a seatbelt alone significantly increases the likelihood of fatal accidents in the case of normal physical and road conditions. As shown in Table 2, the probability of a fatal accident with a seatbelt is 0.259, whereas without a seatbelt, this probability increases dramatically to 4.793. When this absence of a seatbelt is combined with DUI and snowy road conditions, the interactions among these factors lead to a dangerous increase in risk to 5.193.

Introducing a mitigating factor such as seatbelt use offers an opportunity to explore how such measures influence complex risk dynamics. In the case of DUI and snowy road conditions but with the driver wearing a seatbelt, the probability of a fatal accident decreases to 0.297, a significant reduction compared with the fatal accident probability of 5.193 when no seatbelt is used but with DUI and snowy road conditions. Nevertheless, despite this reduction in risk, a small but nonnegligible nonlinear amplification in risk remains. Furthermore, the probability of a fatal accident predicted by the additive model in the case of DUI and snowy road conditions is 0.032, but the probability predicted by the combined effects model for the same conditions is slightly greater at 0.038, reflecting an 18.7% increase. This residual nonlinear effect, even in the case of strong mitigating factors such as seatbelt use, demonstrates that the mitigation of risk with such factors cannot fully cancel out the increase in risk due to interactions among adverse factors. While seatbelt use significantly reduces overall risk, the interaction between DUI and snowy conditions remains important enough to result in a measurable nonlinear increase in risk. This suggests that even when interventions are implemented, some increase in risk persists owing to the inherent interdependencies among risk factors.

#### Introduction to the concept of a synergistic effect

To explain this phenomenon on the basis of the calculations, the individual effects of each risk factor are first examined. For example, DUI increases the probability of a fatal accident from 0.259 (under normal conditions) to 0.277, representing an increase of 0.018. Snowy conditions increase the probability of a fatal accident from 0.259 to 0.273, an increase of 0.014. Finally, not wearing a seatbelt has the largest impact, increasing the probability of a fatal accident from 0.259 to 4.793, an increase of 4.534. If these individual effects were simply added together via a linear model, these three factors (DUI, snowy conditions, and no seatbelt) would result in an "added factor effect" of 4.566. However, when these factors are combined with the proposed BN model, the probability of a fatal accident increases to 5.193, which is significantly greater than the linear sum of the individual effects. The difference between the added factor effect (4.566) and the combined effect (4.934) is 0.368, indicating that the overall risk increases owing to the interaction among the factors rather than their individual contributions.

To quantify the extent of this amplified risk, the nonlinear effect factor is calculated by comparing the combined effect to the added effect. The result is 1.081, which represents an 8.1% increase in risk when these factors are combined. This 8.1% increase in risk demonstrates that the overall risk is greater than that predicted by simply summing the individual risks.

In this context, a synergistic effect refers to the way in which risk factors, such as DUI, snowy conditions, and lack of a seatbelt, interact to amplify the overall danger beyond the simple sum of their individual risks. This synergistic effect arises because the risk factors are not independent; these factors not only increase the risk individually but also influence one another in a manner that results in compounded effects, increasing the overall danger. For example, DUI affects a driver’s ability to react to hazardous conditions such as snow, whereas snowy conditions make the road more dangerous, particularly for an impaired driver. Furthermore, the absence of a seatbelt dramatically increases the likelihood of a fatality if an accident occurs, and DUI on a snowy road further amplifies this risk. Thus, when these factors are all present, the effects of each factor reinforce the effects of the other factors, creating a situation in which the combined danger is much greater than the sum of the individual risks. This is the core idea of a synergistic effect: each factor influences the others, leading to a nonlinear increase in risk that would not be captured if each factor were considered in isolation.

In conclusion, the synergistic effect in this multifactor scenario is a result of the interaction among the DUI driver state, snowy road conditions, and lack of a seatbelt, leading to an increase in the overall risk. The 8.1% increase in risk shows that the total risk exceeds the sum of the individual risks, underscoring the limitations of traditional linear models that consider these risk factors independently. Instead, these interactions create a synergistic effect with the risk of each factor compounding, significantly increasing the likelihood of a fatal accident. This demonstrates the need for models that account for the complex interdependencies among human behaviour, environmental conditions, and safety measures.

#### Policy perspectives related to the road safety scenario

From a policy perspective, the insights from the analysis suggest that a more targeted approach to risk mitigation is necessary. Traditional safety campaigns often focus on individual risk factors, such as DUI enforcement or the promotion of seatbelt use, without fully considering how these factors interact in real-world scenarios. However, the results reveal that the combination of multiple high-risk factors, such as impaired driving, adverse weather conditions, and the absence of safety equipment, can result in a nonlinear increase in danger, far exceeding the risk posed by any single factor. This insight underscores the importance of addressing these complex, interactive risks in a holistic manner.

To be truly effective, mitigation strategies must move beyond isolated interventions and focus on the interactions among risk factors that result in increased danger. For example, enforcing DUI laws more rigorously during adverse weather conditions, when roads are snowy or icy, could significantly reduce the combined risk. The results show that the interactions among these factors lead to a substantial increase in the risk of fatal accidents. Therefore, a policy that targets impaired drivers specifically during poor weather could have a substantial impact on reducing fatalities compared with simply addressing DUI in general or focusing solely on weather-related risks.

Moreover, these insights could inform the development of targeted public safety initiatives, such as implementing enhanced enforcement campaigns during seasons when adverse weather conditions are more likely (e.g., winter months in snowy regions). Increased police presence on the roads during these high-risk periods, combined with public messaging on the dangers of DUI in bad weather, could reduce the likelihood of scenarios involving multiple high-risk factors. Additionally, integrating this knowledge into driver education programs could provide a more comprehensive understanding of risk, ensuring that drivers are aware of not only the importance of seatbelts and avoiding DUI but also how these factors interact with environmental conditions to significantly increase the likelihood of fatal accidents.

In summary, these insights demonstrate that addressing individual risk factors in isolation is insufficient for effective risk mitigation. Policymakers should focus on the interactions among high-risk factors, particularly those that produce nonlinear increases in danger, and implement strategies that account for these complex interactions. By targeting the most dangerous combinations of risk factors, such as impaired driving during adverse weather, and reinforcing that the use of safety equipment alone is not sufficient to fully mitigate these risks, public safety interventions can more effectively reduce the occurrence of fatal accidents.

The insights from this analysis are critical for understanding real-world risks in complex environments involving multiple risk factors. The results show that traditional risk models are insufficient for capturing these dynamics and emphasize the need for nonlinear modelling approaches such as BNs.

## Discussion and conclusion

Traditional risk models, which treat factors as independent and combine their effects in a linear, additive manner, often underestimate the actual dangers present in multifactorial scenarios. This study introduces a general framework for modelling nonlinear risk interactions that was validated with a road safety example. However, this approach can be applied in various fields, addressing the need for a fundamental shift in how risk interactions are modelled and understood in complex systems.

Although the BN model used in this study was previously introduced in^3^, the current study introduces substantial methodological innovations beyond this prior work. Specifically, the rigorous mathematical formalization and quantification of nonlinear interactions through the newly introduced nonlinear effect factor, as well as the explicit generalization of the framework to handle multifactor interactions, represent significant advancements. However, at this stage, the general framework is illustrated within the road safety context, representing a limitation. Future research plans include validating this general framework across additional application domains such as healthcare or environmental management.

This paper presents a rigorous mathematical formalization of nonlinear factor interactions using BNs. At this methodological stage, classical statistical hypothesis tests (e.g. significance tests, p-value, confidence intervals) are not performed because the emphasis remains on methodological rigor and conceptual demonstration. However, future research aimed at empirical validation and practical applications will require formal statistical validations.

The results underscore the nonlinear nature of interactions among risk factors. In particular, in the case of road safety, the interactions among DUI, snowy road conditions, and the absence of a seatbelt were investigated. When all these factors are present, the risk of a fatal accident is significantly increased compared with that predicted by a linear, additive model. The nonlinear effect factor (γ) calculated in this study shows how synergistic effects among risk factors can dramatically increase overall risk levels, highlighting the importance of accounting for such interactions in comprehensive risk assessments. This is a crucial result: in complex environments, risk factors rarely act in isolation, and failing to model their interactions can lead to serious underestimations of the likelihood of adverse outcomes. This observation is true not only in the context of road safety but also in any field in which multiple factors are involved in high-risk situations.

Although this study focuses on road safety, the general framework of modelling nonlinear risk interactions using BNs has applications far beyond this specific domain. This proposed general framework provides a robust approach for understanding and modelling systems in which multiple factors interact in nonlinear, complex ways. By providing a mathematical model based on BNs, this study enables an approach that allows for more accurate analysis of risks across various fields, including public health and environmental management.

The practical implications of this study are significant, especially for risk management and policy development. Decision makers need to recognize the nonlinear nature of risk interactions when designing preventive measures and interventions. For example, policies that target the simultaneous occurrence of multiple high-risk factors, such as DUI during adverse weather, could lead to the development of more effective accident prevention strategies. Moreover, this study shows that seatbelt campaigns, while critical, should be part of a broader risk management strategy that addresses the interplay among multiple risk factors rather than relying solely on one form of mitigation.

The general framework proposed in this study provides a strong foundation for advancing risk analysis across multiple domains. The BN approach inherently incorporates uncertainty quantification through probabilistic reasoning. However, additional uncertainty arises from various sources, such as data quality, measurement errors, incomplete information, and assumptions about conditional dependencies among risk factors. These uncertainties, present in the initial data and hypotheses, propagate explicitly through the BN, influencing the final risk estimates. Future work should investigate how these uncertainties affect the reliability and robustness of results obtained from this framework, potentially comparing these uncertainty effects with traditional linear and alternative nonlinear models. Moreover, future research should focus on empirical comparisons between the proposed framework and alternative advanced methods, including dynamic simulation models^4^.

To further demonstrate the broader applicability of the framework beyond road safety, in the supplementary Information (Appendix D), an additional case study on public health, specifically focusing on type 2 diabetes risk, is included. Using real-world survey data from the U.S. Centers for Disease Control and Prevention (CDC) Behavioral Risk Factor Surveillance System (BRFSS)^38^, the illustration is provided focusing on how clinical, behavioral and socio-demographic factors interact nonlinearly to amplify diabetes risk. The results from this second validation confirm the robustness and cross-domain generalizability of the NFBR framework.

Although the proposed framework effectively captures nonlinear interactions, it has certain limitations. For example, the accuracy of the framework heavily relies on data quality and availability. Additionally, complex networks involving numerous interacting factors may present computational challenges. Another limitation of the current illustration is the temporal scope of the dataset used, which covers a single quarter. Therefore, potential seasonal cyclical effects on risk factor could not be captured^3^. Future empirical validations should include longer observational periods. However, this temporal limitation does not affect the validity or generalizability of the methodological framework and mathematical calculations presented.

## Supplementary Information

Below is the link to the electronic supplementary material.

Supplementary Material 1

## Data Availability

Data availability statement The “road safety” dataset is open source and was published by the Maryland Government (accessed 19.05.2025) (https://mdsp.maryland.gov/Pages/Dashboards/DashboardHome.aspx) or the excel direct link: (https://mdsp.maryland.gov/Documents/CrashData/Crash_2018.xlsx) The “Diabetes” dataset is open source and was published by the U.S. Centers for Disease Control and Prevention (CDC). (accessed 06.06.2025). (https://www.cdc.gov/brfss/annual_data/annual_2023.html).
