# Supplementary material for: General framework of nonlinear factor interactions using bayesian networks for risk analysis applied to road safety and public health
Source: Sci Rep. 2025 Aug 15;15:29934. doi: 10.1038/s41598-025-13572-5 (PMC12356956; doi:10.1038/s41598-025-13572-5)
Supplement: Supplementary file 1 — Supplementary Material 1 [file 41598_2025_13572_MOESM1_ESM.docx]

# Appendix A

Variables of Driving Risk Factors after Discretization

| **Classification** | **Variables** | **Code** | **Description** | **Frequency** | **Percent** |
| --- | --- | --- | --- | --- | --- |
| Human (driver) | Gender | 0 | Unknown | 4256 | 15.9% |
|  |  | 1 | Female | 8838 | 33.0% |
|  |  | 2 | Male | 13652 | 51.0% |
|  |  |  |  |  |  |
|  | Age | 0 | Unknown | 4559 | 17.0% |
|  |  | 1 | Young (15 to 24) | 4455 | 16.7% |
|  |  | 2 | Adult (25 to 65) | 15747 | 58.9% |
|  |  | 3 | Senior (> 66) | 1985 | 7.4% |
|  |  |  |  |  |  |
|  | Physical condition | 100 | Not Applicable | 1887 | 7.1% |
|  |  | 101 | Apparently normal | 19417 | 72.6% |
|  |  | 102 | Influenced by medications, drugs, alcohol | 1335 | 5.0% |
|  |  | 103 | Illness, handicaps | 118 | 0.4% |
|  |  | 104 | Fatigued, fainted | 139 | 0.5% |
|  |  | 105 | Depressed, angry, disturbed | 45 | 0.2% |
|  |  | 106 | Other | 104 | 0.4% |
|  |  | 99 | Unknown | 3701 | 13.8% |
|  |  |  |  |  |  |
| Environment (Road) | Road geometry | 0 | Not Applicable | 8190 | 30.6% |
|  |  | 1 | Non-intersection | 8274 | 30.9% |
|  |  | 2 | Intersection | 6559 | 24.5% |
|  |  | 3 | Intersection related | 2361 | 8.8% |
|  |  | 5.01 | Interchange related | 467 | 1.8% |
|  |  | 6.01 | Crossover related | 123 | 0.5% |
|  |  | 7.01 | Railway grade crossing | 5 | 0.0% |
|  |  | 8.04 | Residential driveway | 114 | 0.4% |
|  |  | 9.04 | Commercial driveway | 259 | 1.0% |
|  |  | 10.04 | Alley | 16 | 0.1% |
|  |  | 88 | Other | 342 | 1.3% |
|  |  | 99 | Unknown | 36 | 0.1% |
|  |  |  |  |  |  |
|  | Surface condition | 100 | Not Applicable | 3515 | 13.1% |
|  |  | 101 | Wet | 4957 | 18.5% |
|  |  | 102 | Dry | 15621 | 58.4% |
|  |  | 103 | Snow, Ice, slush | 2390 | 9.0% |
|  |  | 104 | Other | 62 | 0.2% |
|  |  | 99 | Unknown | 201 | 0.8% |
|  |  |  |  |  |  |
| Environment | Weather | 100 | Not Applicable | 2795 | 10.5% |
|  |  | 101 | Foggy | 267 | 1.0% |
|  |  | 102 | Raining | 3094 | 11.6% |
|  |  | 103 | Severe winds | 303 | 1.1% |
|  |  | 104 | Clear | 15180 | 56.7% |
|  |  | 105 | Cloudy | 2575 | 9.6% |
|  |  | 106 | Snow, sleet, wintry mix | 2145 | 8.0% |
|  |  | 107 | Other | 157 | 0.6% |
|  |  | 99 | Unknown | 230 | 0.9% |
|  |  |  |  |  |  |
|  | Light condition | 0 | Not Applicable | 415 | 1.5% |
|  |  | 1 | Daylight | 14625 | 54.7% |
|  |  | 3 | Dark lights on | 6999 | 26.2% |
|  |  | 4 | Dark no lights | 2298 | 8.6% |
|  |  | 5.02 | Dawn | 769 | 2.9% |
|  |  | 6.02 | Dusk | 720 | 2.7% |
|  |  | 7.88 | Dark-unknown lighting | 409 | 1.5% |
|  |  | 88 | Other | 78 | 0.3% |
|  |  | 99 | Unknown | 433 | 1.6% |
|  |  |  |  |  |  |
| Vehicle | Type | 100 | Not Applicable | 79 | 0.3% |
|  |  | 101 | Motorcycles | 79 | 0.3% |
|  |  | 102 | Cars | 19324 | 72.3% |
|  |  | 103 | Trucks | 536 | 2.0% |
|  |  | 104 | Bus | 463 | 1.7% |
|  |  | 105 | Service vehicles | 646 | 2.4% |
|  |  | 106 | Vans and Pickup | 2884 | 10.8% |
|  |  | 107 | Others | 403 | 1.5% |
|  |  | 99 | Unknown | 2332 | 8.7% |
|  |  |  |  |  |  |
|  | Safety equipment | 100 | Not Applicable | 1749 | 6.6% |
|  |  | 101 | None | 515 | 1.9% |
|  |  | 102 | Belt | 19024 | 71.1% |
|  |  | 103 | Other | 49 | 0.2% |
|  |  | 104 | MC/bike helmet, protective pads, reflective clothing | 60 | 0.2% |
|  |  | 99 | Unknown | 5349 | 20.0% |
|  |  |  |  |  |  |
|  | Movement | 0 | Not Applicable | 230 | 0.9% |
|  |  | 1 | Moving constant speed | 11858 | 44.3% |
|  |  | 2 | Accelerating | 2190 | 8.2% |
|  |  | 3 | Slowing or stopping | 2888 | 10.8% |
|  |  | 4 | Starting from lane | 559 | 2.1% |
|  |  | 5 | Starting from parked | 384 | 1.4% |
|  |  | 6 | Stopped in traffic lane | 158 | 0.6% |
|  |  | 7 | Changing lanes | 927 | 3.5% |
|  |  | 8 | Passing | 211 | 0.8% |
|  |  | 9 | Parking | 367 | 1.4% |
|  |  | 10 | Parked | 130 | 0.5% |
|  |  | 11 | Backing | 1145 | 4.3% |
|  |  | 12 | Making left turn | 2266 | 8.5% |
|  |  | 13 | Making right turn | 695 | 2.6% |
|  |  | 14 | Right turn on red | 33 | 0.1% |
|  |  | 15 | Making U turn | 213 | 0.8% |
|  |  | 16 | Skidding | 541 | 2.0% |
|  |  | 17 | Driverless moving vehicle | 8 | 0.0% |
|  |  | 18.07 | Leaving traffic lane | 110 | 0.4% |
|  |  | 19.07 | Entering traffic lane | 151 | 0.6% |
|  |  | 20.03 | Negotiating a curve | 200 | 0.7% |
|  |  | 88 | Other | 141 | 0.5% |
|  |  | 99 | Unknown | 1341 | 5.0% |
|  |  |  |  |  |  |
| Collision | Collision type | 0 | Not Applicable | 245 | 0.9% |
|  |  | 1 | Head on | 615 | 2.3% |
|  |  | 2 | Head on left turn | 1214 | 4.6% |
|  |  | 3 | Same direction rear end | 6794 | 25.4% |
|  |  | 4 | Same direction rear end right turn | 86 | 0.3% |
|  |  | 5 | Same direction rear end left turn | 133 | 0.5% |
|  |  | 6 | Opposite direction sideswipe | 335 | 1.3% |
|  |  | 7 | Same direction sideswipe | 1871 | 7.0% |
|  |  | 8 | Same direction right turn | 542 | 2.0% |
|  |  | 9 | Same direction left turn | 545 | 2.0% |
|  |  | 10 | Same direction both left turn | 118 | 0.5% |
|  |  | 11 | Same movement angle | 3423 | 12.8% |
|  |  | 12 | Angle meets right turn | 114 | 0.4% |
|  |  | 13 | Angle meets left turn | 134 | 0.5% |
|  |  | 14 | Angle meets left turn head on | 75 | 0.3% |
|  |  | 15 | Opposite direction both left turn | 53 | 0.2% |
|  |  | 17 | Single vehicle | 6473 | 24.2% |
|  |  | 88 | Other | 3331 | 12.4% |
|  |  | 99 | Unknown | 645 | 2.4% |
|  |  |  |  |  |  |
| Risk | Crash severity | 1 | Fatal crashes | 87 | 0.4% |
|  |  | 2 | Injury crashes | 7393 | 27.6% |
|  |  | 3 | Property damage only | 19266 | 72.0% |
|  |  |  | TOTAL crash reports | 26746 | *100.0%* |

# Appendix B

Scenario #1– column A


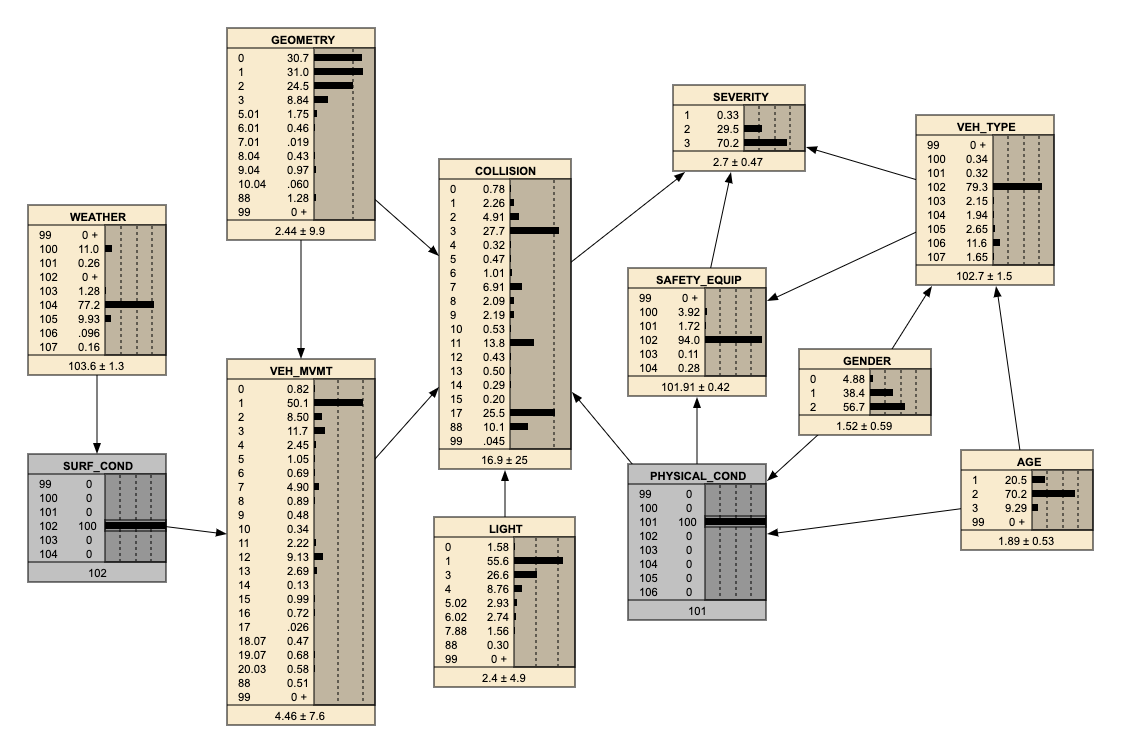


Scenario #1 – column B


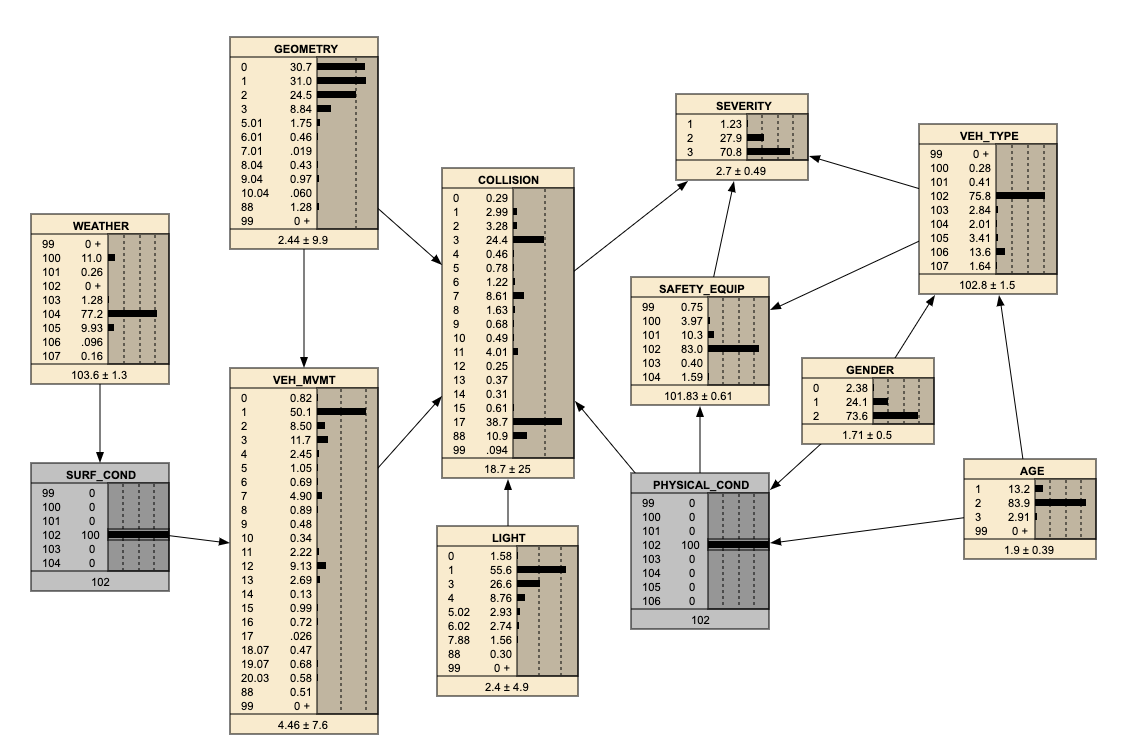


Scenario #1– column C


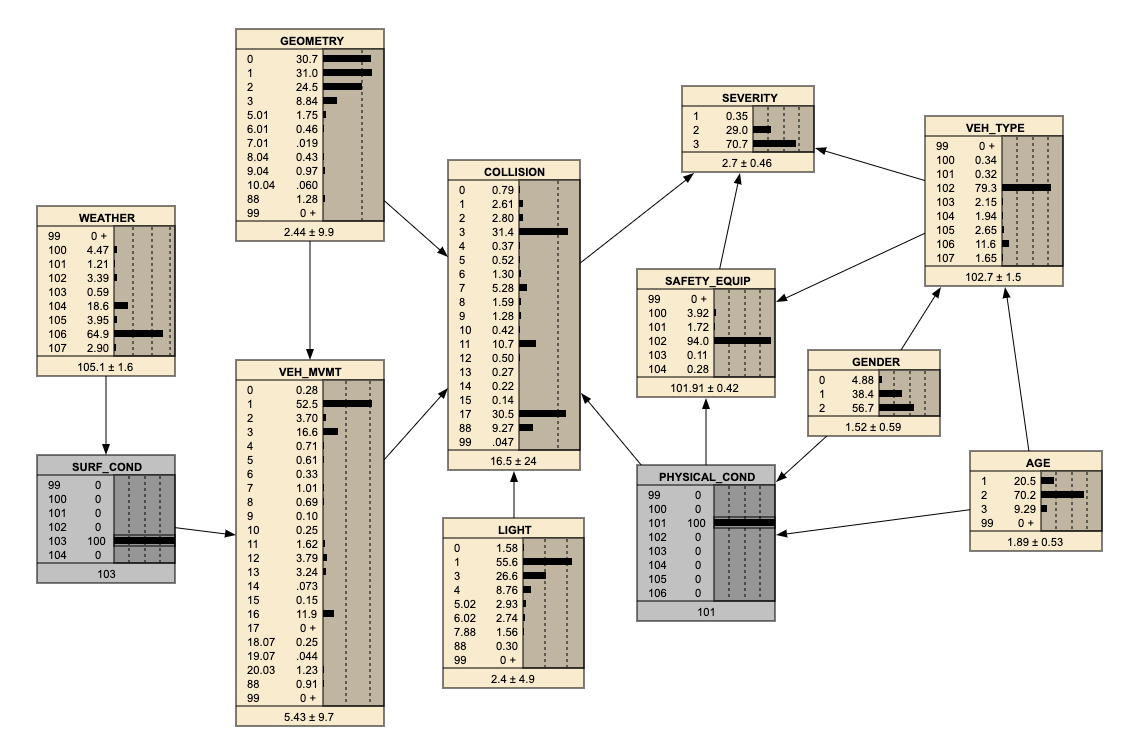


Scenario #1– column D


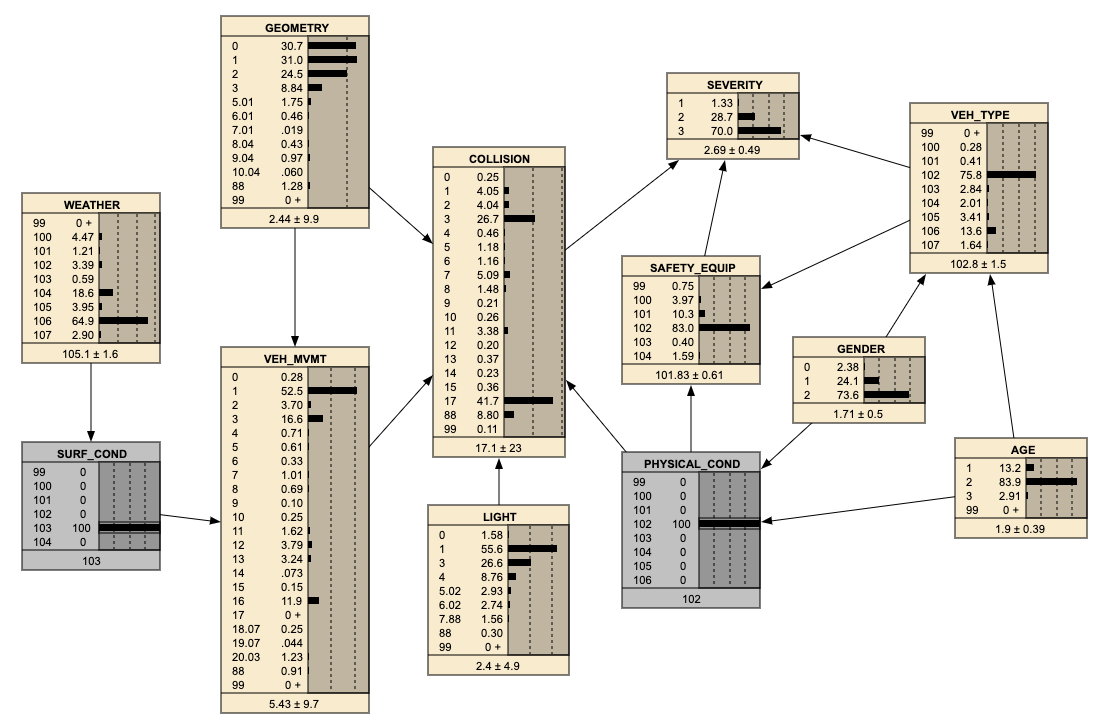


# Appendix C

Scenario #3– column A


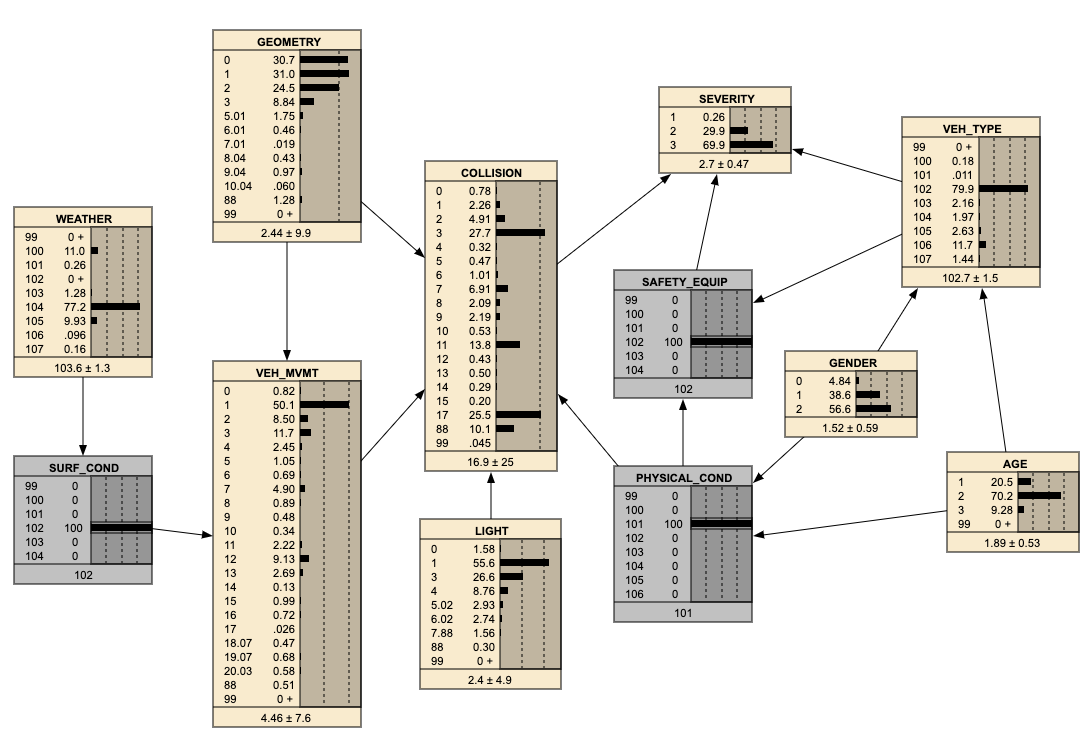


Scenario #3– column B


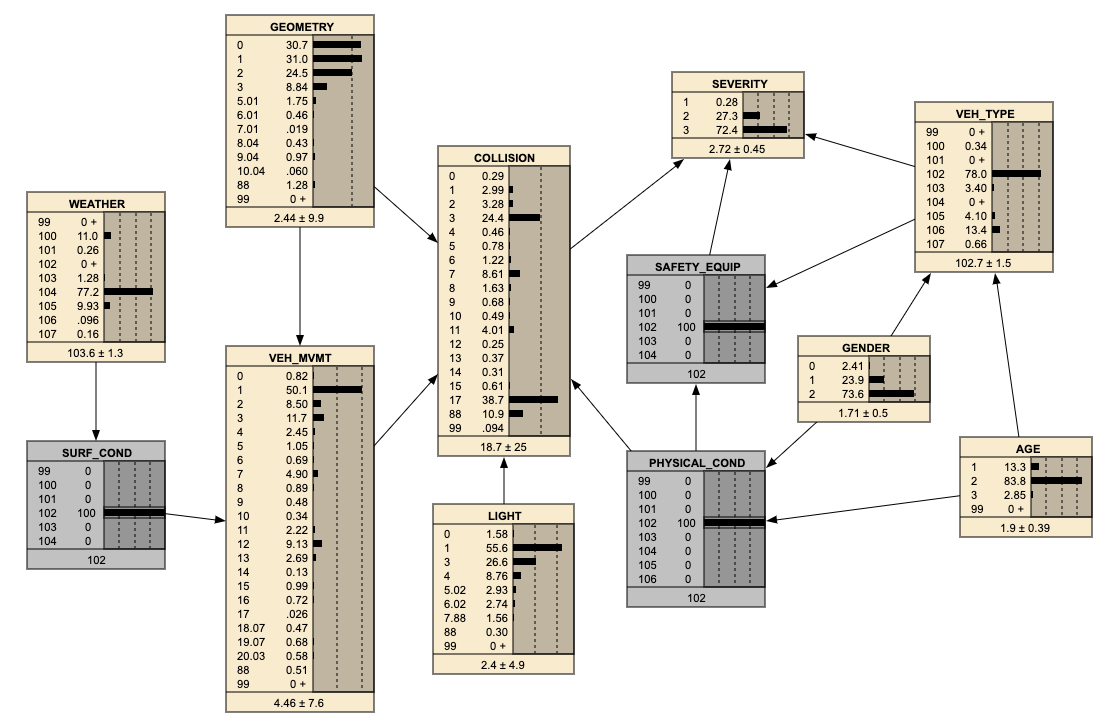


Scenario #3– column C


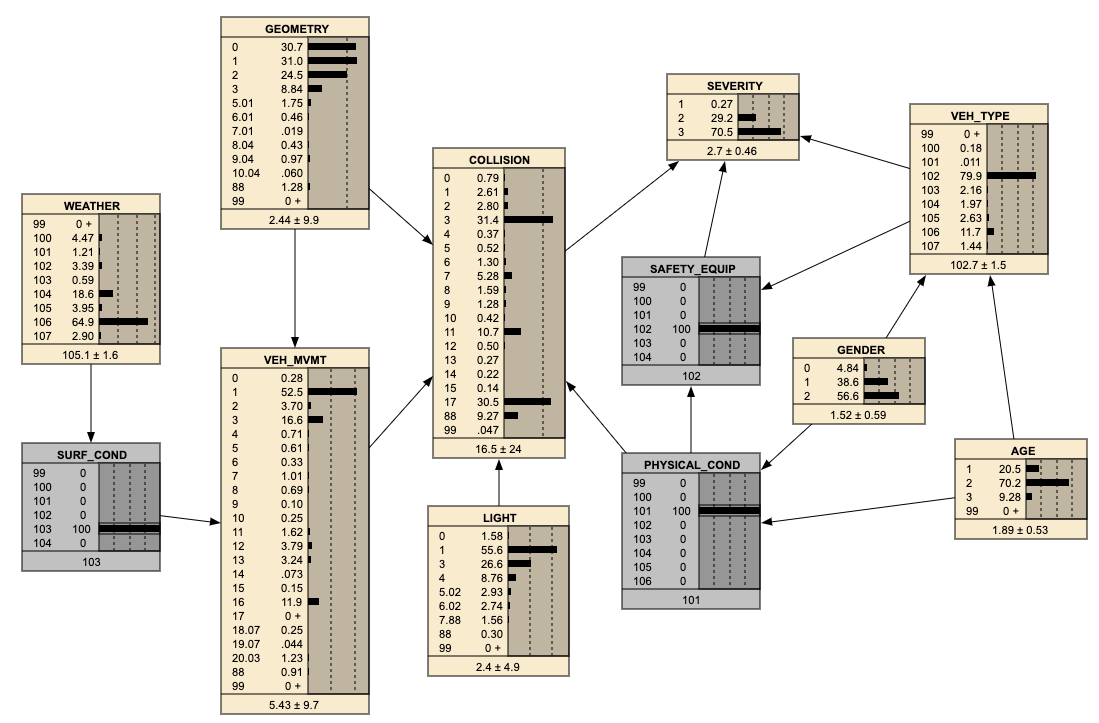


Scenario #3– column D


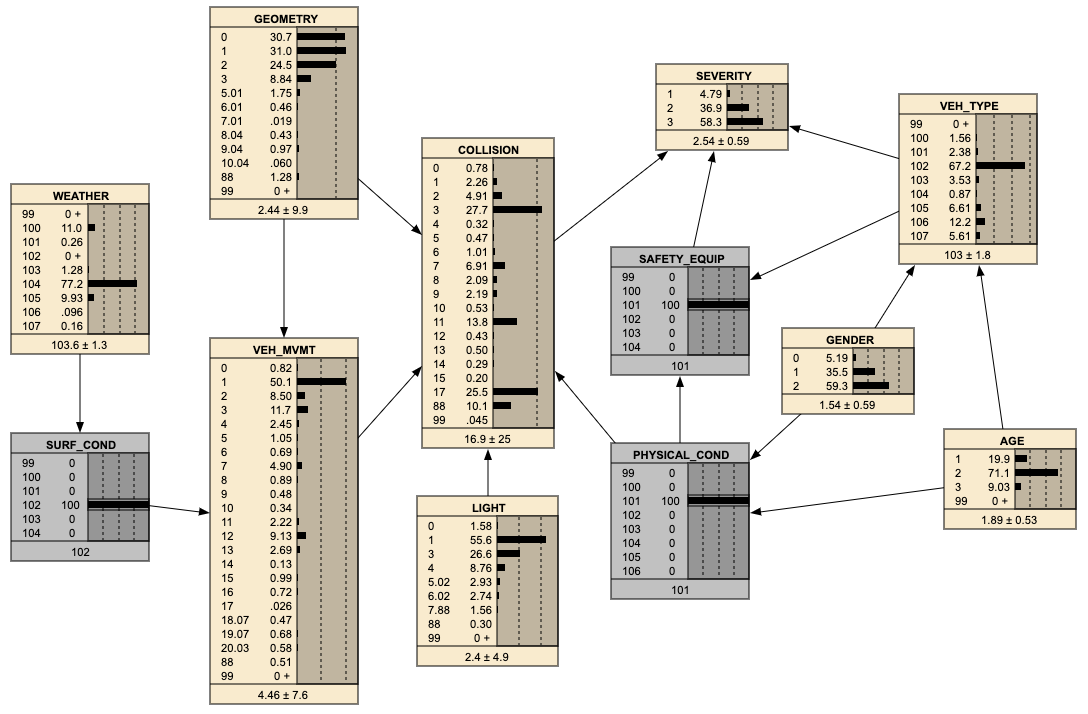


Scenario #3– column E


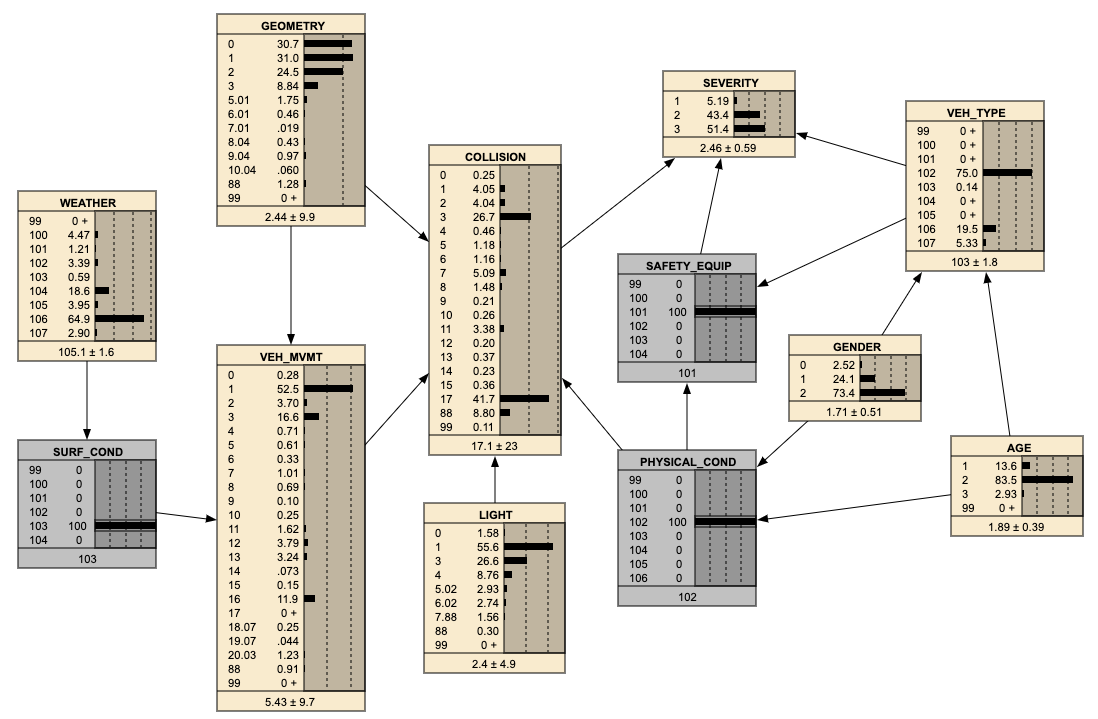


Scenario #3– column G


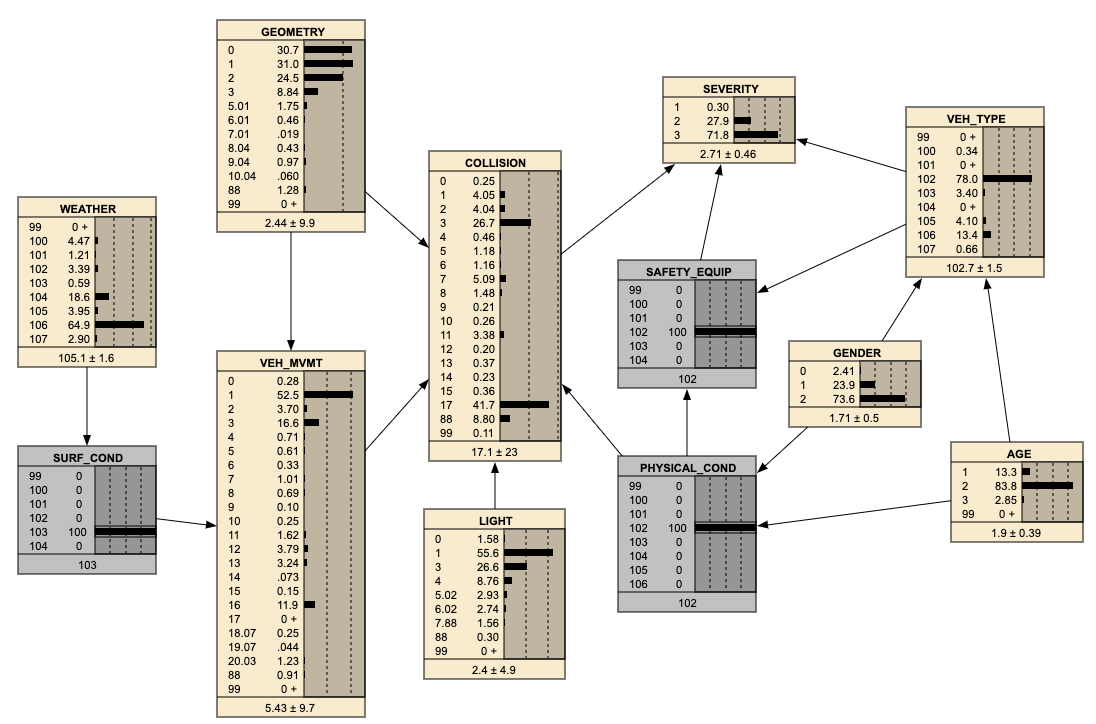


# Appendix D

# CASE STUDY: Risk of Diabetes (T2)

# This annex illustrates the application of the proposed NFBR framework in the domain of public health. It focuses on type 2 diabetes, a chronic disease influenced by multiple interacting risk factors.

# The objective of this study is to model an quantify how these factors contribute to diabetes risk in a nonlinear way.

# 1.METHODOLOGY

# The process followed the general framework of NFBR. We began with the identification of risk factors and the selection of relevant variables based on literature review and the U.S. Centers for Disease Control and Prevention (CDC) recommendations.

# The Bayesian network model was developed in two steps:

# Structure learning was performed using a score-based approach (Tabu Search algorithm with Bayesian information criterion, BIC) implemented in the “bnlearn” package [1] originally developed for R and implemented in the Python version.

# Parameter learning was conducted using Expectation-Maximization (EM) algorithm with Netica software [2], which enables robust estimation of conditional probability tables (CPTs), including cases with missing or unknown values.

# The model was evaluated with a sensitivity analysis, assessing how variations in input variables influence the posterior probability of diabetes outcomes.

# Finally, we have computed the nonlinear effect factor for two scenarios, followed by a brief analysis:

# Scenario 1: Interaction between high blood pressure and no physical activities (two risk factors)

# Scenario 2: Interaction between high blood pressure, no physical activities and smoking (three risk factors)

# 2.DATA SOURCE AND VARIABLES SELECTION

The diabetes case study presented uses real-world data from the 2023 Behavioral Risk Factor Surveillance System (BRFSS), a large-scale health survey conducted annually by the CDC in the United States [3]. The BRFSS collects information on health-related risk behaviors, chronic health conditions, and use of preventive services across all U.S. states. The dataset is survey-based and includes responses from adults across 48 U.S. States.

**Source:** U.S. Centers for Disease Control and Prevention (CDC). (2025). *Behavioral Risk Factor Surveillance System: 2023 BRFSS Data*. Available: <https://www.cdc.gov/brfss/annual_data/annual_2023.html>. [Accessed: 6.06.2025].

Variable definitions and coding follow the BRFSS 2023 Codebook provided by CDC.

The selection of variables for the diabetes BN model was guided by established risk factors reported in the literature and their availability in the BRFSS dataset. Clinical (e.g., blood pressure, BMI), behavioral (e.g., smoking, alcohol use), and socio-demographic (e.g., age, education) dimensions were included to reflect multifactorial determinants of type 2 diabetes.

# Variables are discretized into clinically meaningful categories, based literature, such as BMI categorized as Underweight, Normal, Overweight and Obese. The other variables, kept the same categories as the original dataset from CDC.

# Missing or unknown values are kept and reallocated in the Parameter learning using EM Algorithm.

# The final Dataset included 429’086 observations and 12 variables, detailed hereafter.

# Outcome variable: Diabetes Risk (Diabetes, pre-diabetes, No diabetes)

# Variables:

# Socio-demographic variables (3 vars): Gender, Age, Education level

# Clinical variables (3 vars): Blood pressure, Cholesterol, BMI (Body Mass Index)

# Behavioral variables (3 vars): Smoking status, Heavy alcohol consumption, Physical activities

# General health variables (2 vars): General health status, Difficulty walking or climbing stairs

Variables of Diabetes Risk Factors after Discretization

| **Classification** | **Variables** | **Code** | **Description** | **Frequency** | **Percent** |
| --- | --- | --- | --- | --- | --- |
| Socio-demographic | Gender | 0 | Female | 225’835 | 52.6% |
|  |  | 1 | Male | 203’251 | 47.4% |
|  |  |  |  |  |  |
|  | Age | 1 | 18 to 24 | 26’096 | 6.1% |
|  |  | 2 | 25 to 29 | 20’986 | 4.9% |
|  |  | 3 | 30 to 34 | 24’371 | 5.7% |
|  |  | 4 | 35 to 39 | 26’551 | 6.2% |
|  |  | 5 | 40 to 44 | 27’964 | 6.5% |
|  |  | 6 | 45 to 49 | 26’698 | 6.2% |
|  |  | 7 | 50 to 54 | 30’951 | 7.2% |
|  |  | 8 | 55 to 59 | 33’929 | 7.9% |
|  |  | 9 | 60 to 64 | 41’718 | 9.7% |
|  |  | 10 | 65 to 69 | 45’844 | 10.7% |
|  |  | 11 | 70 to 74 | 43’347 | 10.1% |
|  |  | 12 | 75 to 79 | 34’400 | 8.0% |
|  |  | 13 | 80 or older | 38’692 | 9.0% |
|  |  | * | Unknown | 7’539 | 1.8% |
|  |  |  |  |  |  |
|  | Education | 1 | Never or kindergarten | 671 | 0.2% |
|  |  | 2 | Grades 1 to 8 (Elementary) | 8’193 | 1.9% |
|  |  | 3 | Grades 9 to 11 (Some high school) | 15’971 | 3.7% |
|  |  | 4 | Grade 12 or GED (High school graduate) | 105’688 | 24.6% |
|  |  | 5 | College 1 to 3 years | 113’198 | 26.4% |
|  |  | 6 | College 4 years or more | 183’179 | 42.7% |
|  |  | * | Unknown | 2’186 | 0.5% |
| Clinical | BP High | 1 | Yes | 174’979 | 40.8% |
|  |  | 3 | No | 245’413 | 57.2% |
|  |  | * | Unknown | 8’694 | 2.0% |
|  |  |  |  |  |  |
|  | Chol High | 1 | No | 217’225 | 50.6% |
|  |  | 2 | Yes | 157’689 | 36.7% |
|  |  | * | Unknown | 54’172 | 12.6% |
|  |  |  |  |  |  |
|  | BMI | 0 | Underweight (< 18.5) | 6’710 | 1.6% |
|  |  | 1 | Normal (18.5 $\leq$ BMI < 25) | 115’991 | 27.0% |
|  |  | 2 | Overweight (25 $\leq$ BMI < 30) | 138’430 | 32.3% |
|  |  | 3 | Obese ($\geq$ 30) | 128’177 | 29.9% |
|  |  | * | Unknown | 39’778 | 9.3% |
| Behavior | Smoke | 1 | Yes | 157’373 | 36.7% |
|  |  | 2 | No | 249’487 | 58.1% |
|  |  | * | Unknown | 22’226 | 5.2% |
|  | Alcohol (heavy consumption) | 1 | No | 373’213 | 87.0% |
|  |  | 2 | Yes | 23’510 | 5.5% |
|  |  | * | Unknown | 32’363 | 7.5% |
|  |  |  |  |  |  |
|  | Phys. activities | 1 | Yes | 322’134 | 75.1% |
|  |  | 2 | No | 105’762 | 24.6% |
|  |  | * | Unknown | 1’190 | 0.3% |
| General Health | Gen. Health | 1 | Excellent | 62’882 | 14.7% |
|  |  | 2 | Very good | 140’869 | 32.8% |
|  |  | 3 | Good | 142’671 | 33.2% |
|  |  | 4 | Fair | 61’289 | 14.3% |
|  |  | 5 | Poor | 20’157 | 4.7% |
|  |  | * | Unknown | 1’218 | 0.3% |
|  |  |  |  |  |  |
|  | Difficulty to walk | 1 | Yes | 66’415 | 15.5% |
|  |  | 2 | No | 345’136 | 80.4% |
|  |  | * | Unknown | 17’535 | 4.1% |
| Risk | Diabetes | 1 | Diabetes | 59’786 | 13.9% |
|  |  | 3 | No Diabetes | 358’706 | 83.6% |
|  |  | 4 | Pre-diabetes | 10’594 | 2.5% |
|  |  |  | TOTAL | 429’086 | *100.0%* |

1. **RESULTS**
   1. **Bayesian Network Model – Diabetes case study**


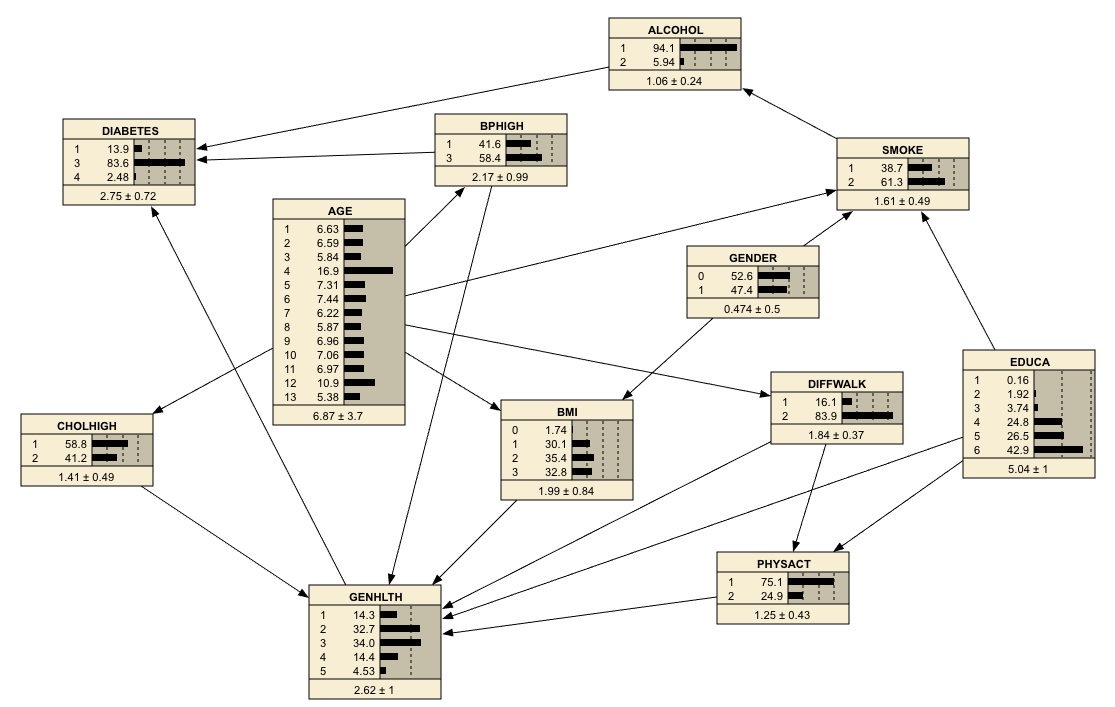


- 1. **BN Model validation – sensitivity analysis**

A sensitivity analysis, conducted in Netica, quantifies the influence of each variable on the Diabetes outcome node. The results based on mutual information show that high blood pressure (5.73%), General health status (5.32%) and Age (3.42%) are the most influential predictors (Table 4). Variables, such as Difficulty to walk, high cholesterol and BMI have a moderate effect, while the others have lower effects.

These findings are consistent with literature [4, 5], were clinical and physiological variables are considered primary predictors of type 2 diabetes risk.

| **Node** | **Mutual Information** | **Percent** | **Variance of Beliefs** |
| --- | --- | --- | --- |
| DIABETES | 0.51600 | 100.00000 | 0.1488603 |
| BPHIGH | 0.02957 | 5.73000 | 0.0093995 |
| GENHLTH | 0.02746 | 5.32000 | 0.0087780 |
| AGE | 0.01766 | 3.42000 | 0.0056271 |
| DIFFWALK | 0.00805 | 1.56000 | 0.0024686 |
| CHOLHIGH | 0.00375 | 0.72700 | 0.0012057 |
| BMI | 0.00265 | 0.51500 | 0.0008693 |
| PHYACT | 0.00203 | 0.39400 | 0.0006413 |
| ALCOHOL | 0.00123 | 0.23800 | 0.0003758 |
| EDUCA | 0.00052 | 0.10100 | 0.0001715 |
| SMOKE | 0.00041 | 0.07960 | 0.0001338 |
| GENDER | 0.00000 | 0.00000 | 0.0000001 |

**Table 4.** Sensitivity analysis of the Diabetes node

- 1. **Nonlinear effect factor**

| Scenario #1  BP High / No phys. Act.  Node | Code | Description | A  Normal situation | B  BP High | C  No phys. activities | D  Combined factors | E*(computed)*  Added factors |
| --- | --- | --- | --- | --- | --- | --- | --- |
| Diabetes | 1  4  3 | **Diabetes**  Pre-diabetes  No diabetes | **5.648**  1.600  92.752 | **22.838**  3.458  73.704 | **8.208**  2.019  89.773 | **29.210**  3.876  66.914 |  |
| Blood pressure (BP) | 3  1 | Normal  High | 100 | 100 | 100 | 100 |  |
| Phys. activities | 1  2 | Yes  No | 100 | 100 | 100 | 100 |  |
|  |  | Blood pressure  Phys. Activities  **Diabetes Risk** | Normal  Yes  **5.648** | High  Yes  **22.838** | Normal  No  **8.208** | High  No  **29.210** | High  No  **25.398** |
|  |  | *Delta vs. normal sit (A)* |  | ***17.190*** | ***2.560*** | ***23.562*** | ***19.750*** |

**Table 5.** Nonlinear risk factor results for Scenario #1 (BP High and No physical activities) – Diabetes risk

| $Nonlinear effect factor= \gamma=\frac{\Delta(Combined effect)}{\Delta(Additive effect)}=\frac{23.562}{19.750}=1.193$ | (15) |
| --- | --- |

| $\Delta P\left( accident \vert X_{1}, X_{2} \right)=\gamma\cdot\left( \Delta P\left( accident \vert X_{1} \right)+\Delta P\left( accident \vert X_{2} \right) \right)$  $=1.193\cdot\left( 17.190+2.560 \right)=23.562$ | (16) |
| --- | --- |

| Scenario #2  BP High/No phys. act. /  Smoke  Node | Code | Description | A  Normal situation | B  BP High | C  No phys. Act. | D  Smoke | E  Combined 3 factors | F  Added factors | G  Combined 2 factors and no smoke |
| --- | --- | --- | --- | --- | --- | --- | --- | --- | --- |
| Diabetes | 1  4  3 | **Diabetes**  Pre-diabetes  No diabetes | **5.566**  1.586  92.848 | **22.773**  3.450  73.777 | **8.056**  1.999  89.945 | **5.810**  1.629  92.561 | **29.220**  3.890  66.890 |  | **29.200**  3.863  66.937 |
| Blood pressure (BP) | 3  1 | Normal  High | 100 | 100 | 100 | 100 | 100 |  | 100 |
| Phys. activities | 1  2 | Yes  No | 100 | 100 | 100 | 100 | 100 |  | 100 |
| Smoke | 2  1 | No  Yes | 100 | 100 | 100 | 100 | 100 |  | 100 |
|  |  | Blood press.  Phys. Act.  Smoke  **Diabetes Risk** | Normal  Yes  No  **5.566** | High  Yes  No  **22.773** | Normal  No  No  **8.056** | Normal  Yes  Yes  **5.810** | High  No  Yes  **29.220** | High  No  Yes | High  No  No  **29.200** |
|  |  | *Delta vs. normal sit (A)* |  | *17.207* | *2.490* | *0.244* | ***23.654*** | ***19.941*** | ***23.634*** |

**Table 6.** Nonlinear risk factor results for Scenario #2 (BP High, No physical activities and Smoke) – Multifactor interactions for Diabetes risk

| $Nonlinear effect factor= \gamma=\frac{\Delta(Combined effect)}{\Delta(Additive effect)}=\frac{23.654}{19.941}=1.186$ | (17) |
| --- | --- |
| $\Delta P\left( accident \vert X_{1},X_{2},X_{3} \right)=\gamma\cdot\left( \Delta P\left( accident \vert X_{1} \right)+\Delta P\left( accident \vert X_{2} \right)+\Delta P\left( accident \vert X_{3} \right) \right)=1.186\cdot\left( 17.207+2.490+0.244 \right)=23.654$ | (18) |

Scenario #2 - Detailed calculation:

|  | Diabetes Risk |  | 3 Factors variation |  | 2 Factors variation and no smoke |
| --- | --- | --- | --- | --- | --- |
| Risk in normal situations  Risk if BP High  Risk if no physical activities  Risk if smoke | 5.566  22.773  8.056  5.810 | *PB High*  *No act.*  *Smoke* | --  17.207  2.490  0.244 | *BP High*  *No act.*  *Smoke* | --  17.207  2.490 |
| Added factor effect (a)  Combined factor effect (b) | 29.220 |  | 19.941  23.654 |  | 19.697  23.634 |
| Difference (a) – (b) |  |  | **-3.713** |  | **-3.937** |
| Nonlinear effect factor  *(delta-based method)* |  |  |  |  |  |
| Delta combined factor effect  Delta added factor effect |  |  | 23.654  19.941 |  | 23.634  19.697 |
| *Delta combined factor effect / delta added factor effect*  *Percentages* |  |  | ***1.186***  ***18.6%*** |  | ***1.200***  ***20.0%*** |

**Table 7.** Nonlinear effect factor calculated for 2 and 3 risk factors (Diabetes risk)

- 1. **Analysis**

Scenario#1 demonstrates that the combination of high blood pressure and no physical activities leads to a higher increase in diabetes risk compared to their individual effects. While the additive effect (relative to the normal situation) results in an increased risk of 19.75%, the combined effect shows a 23.56% risk increase. The nonlinear effect factor (1.193) indicates a 19.30% amplification beyond the additive model.

Scenario#2 which includes three interacting risk factors, smoking, high blood pressure and no physical activities, reveals a combined diabetes risk increase of 23.65%, compared to the additive risk increase of 19.94%. The nonlinear effect factor (1.186) implies that the combined effect is 18.60% higher than what would be predicted by summing individual effects.

Scenario #1 Diabetes – column A


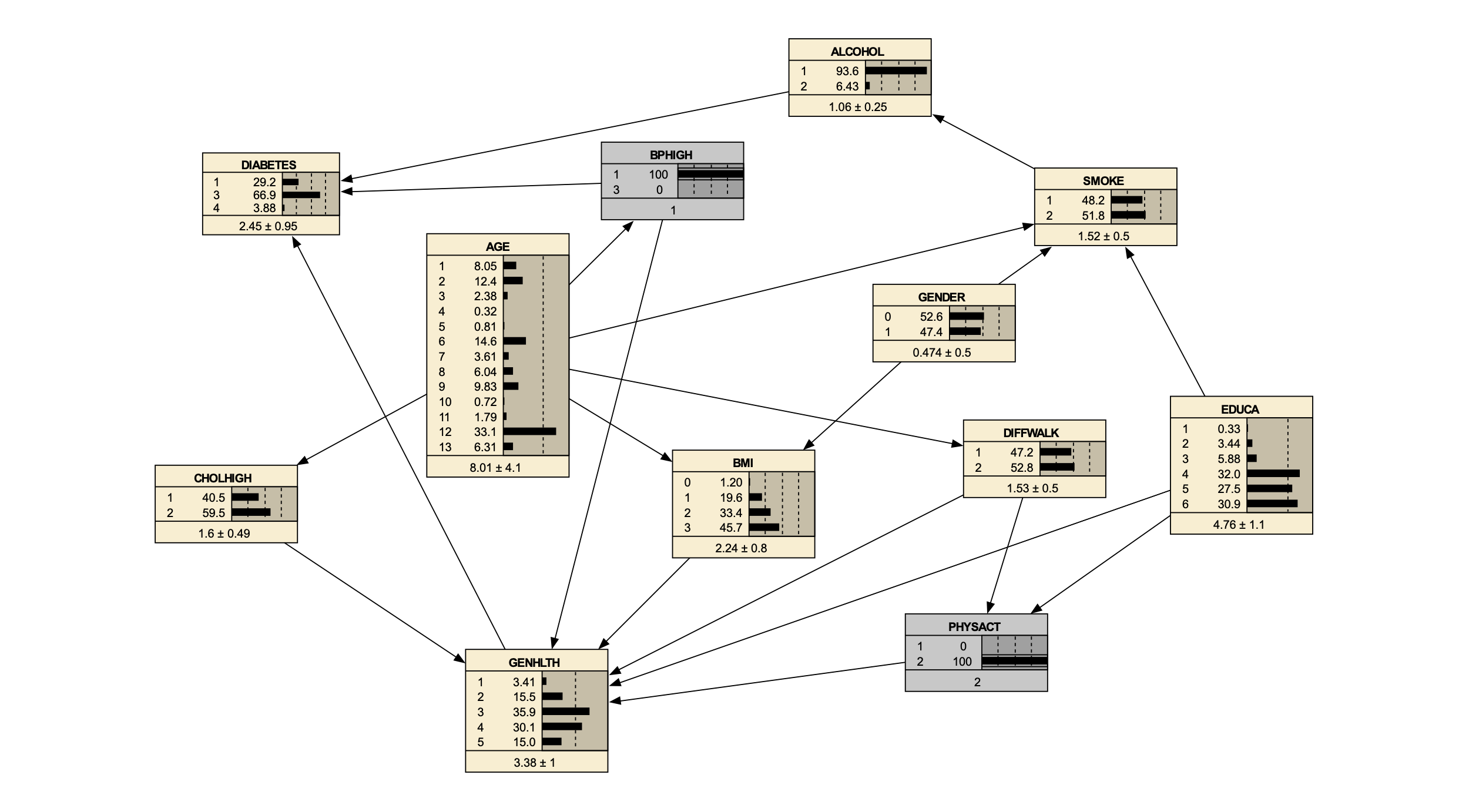


Scenario #1 Diabetes – column B


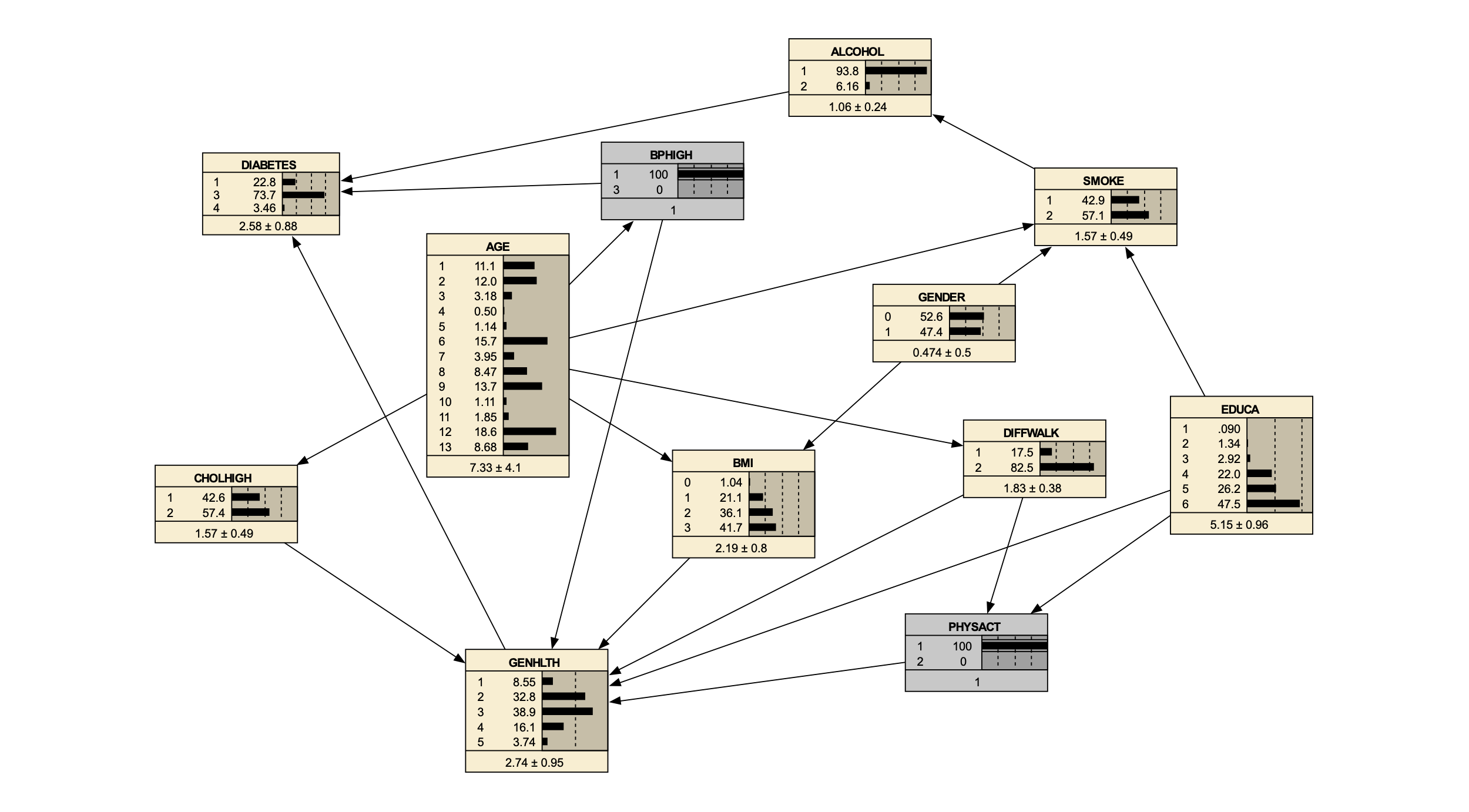


Scenario #1 Diabetes – column C


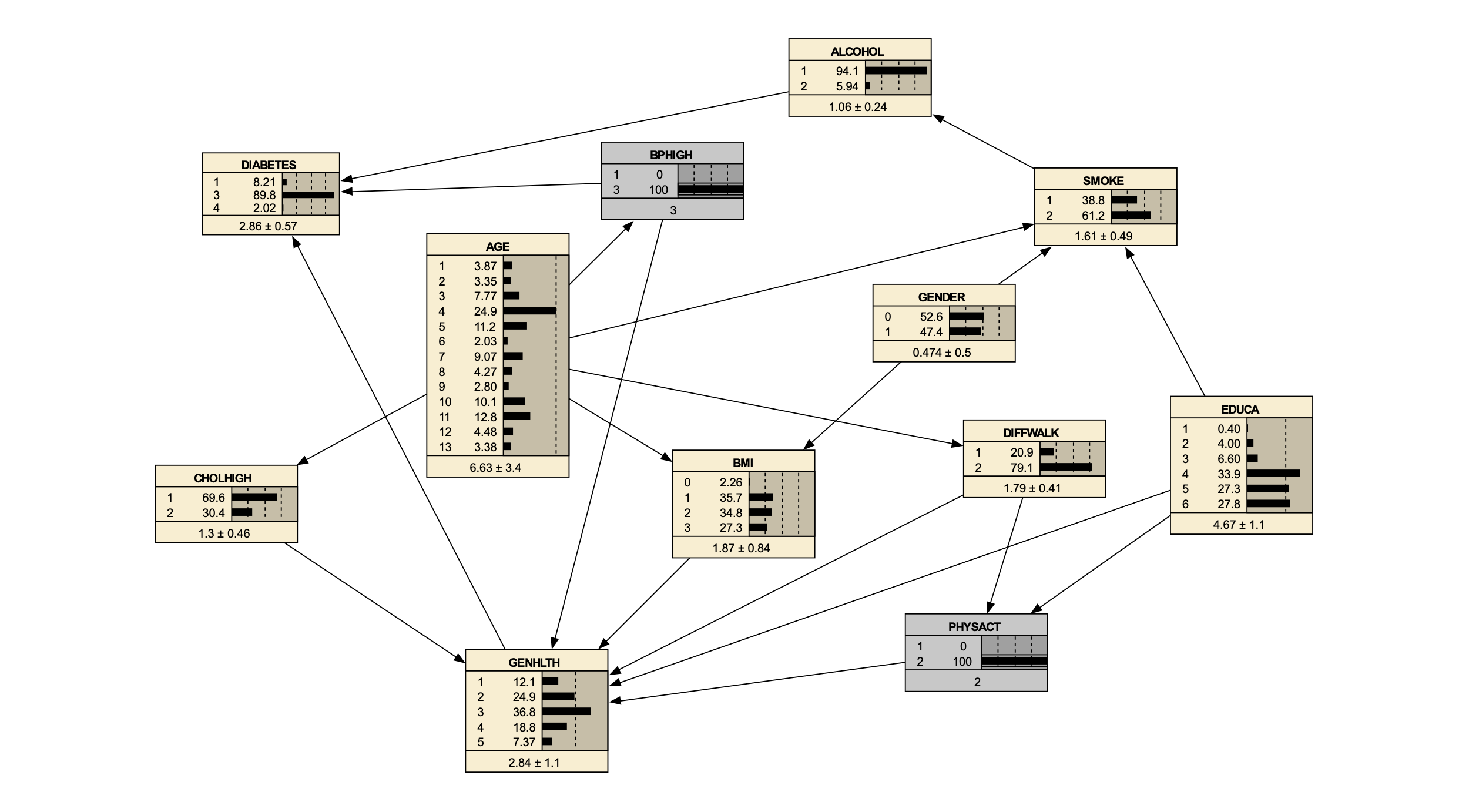


Scenario #1 Diabetes – column D


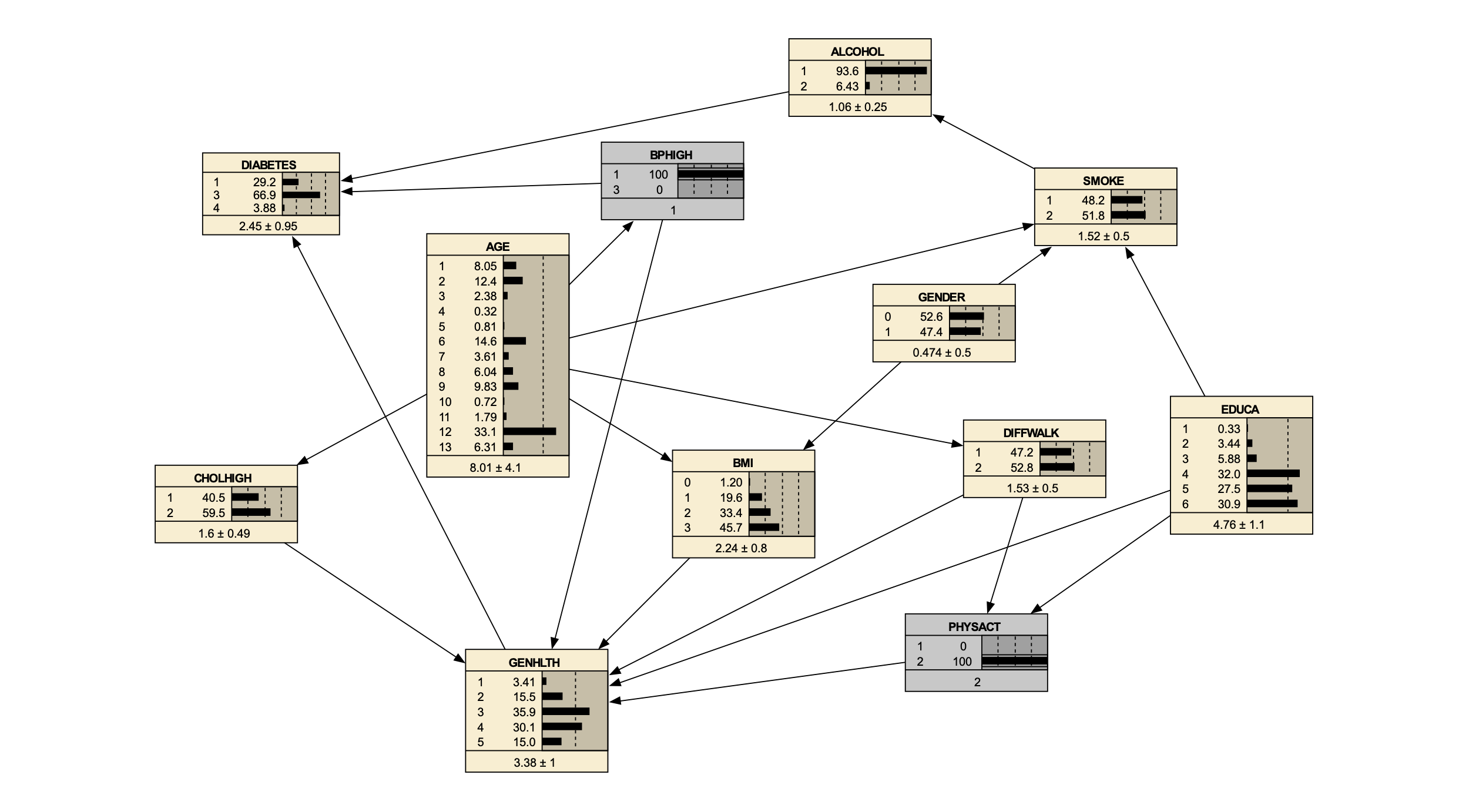


Scenario #2 Diabetes – column A


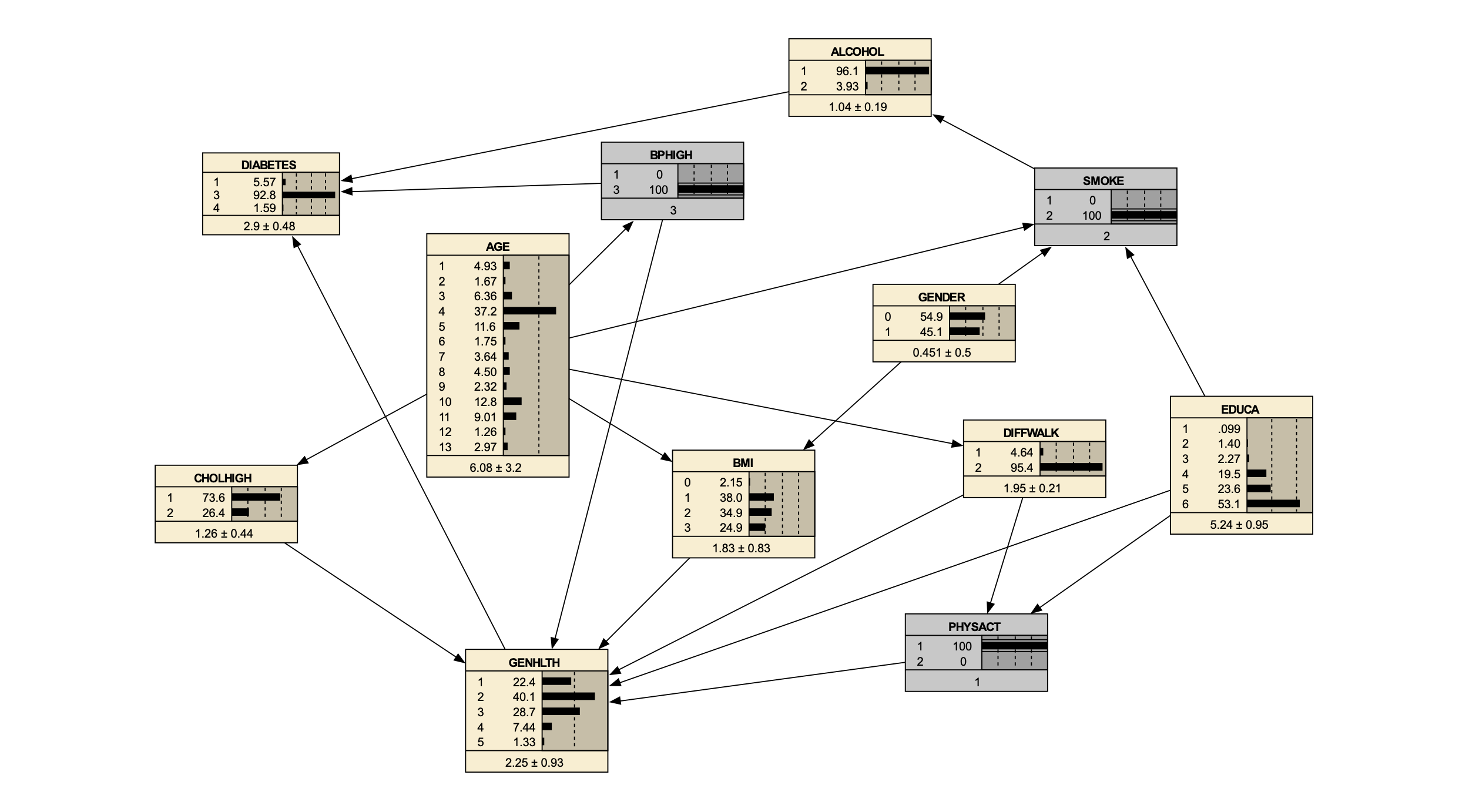


Scenario #2 Diabetes – column B


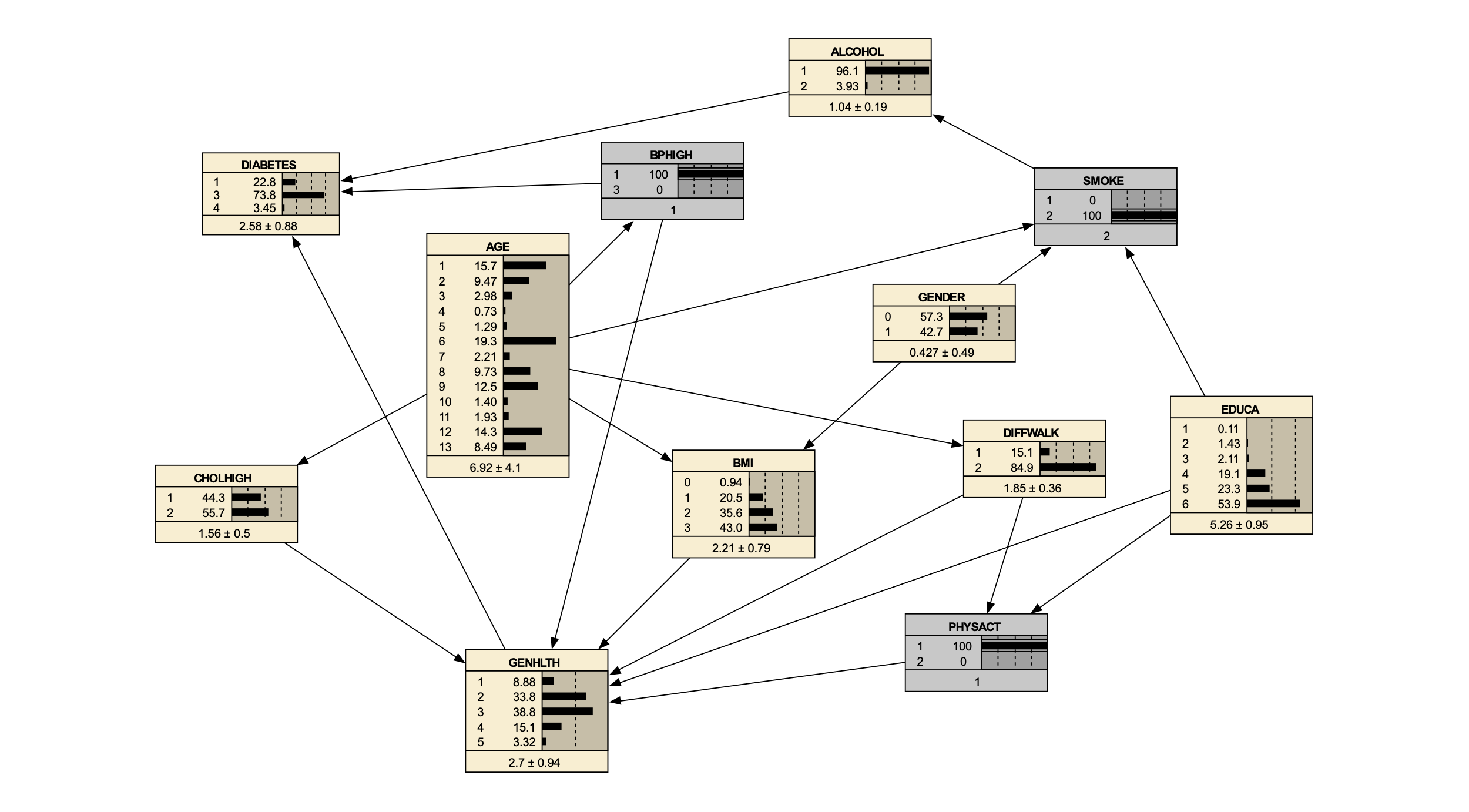


Scenario #2 Diabetes – column C


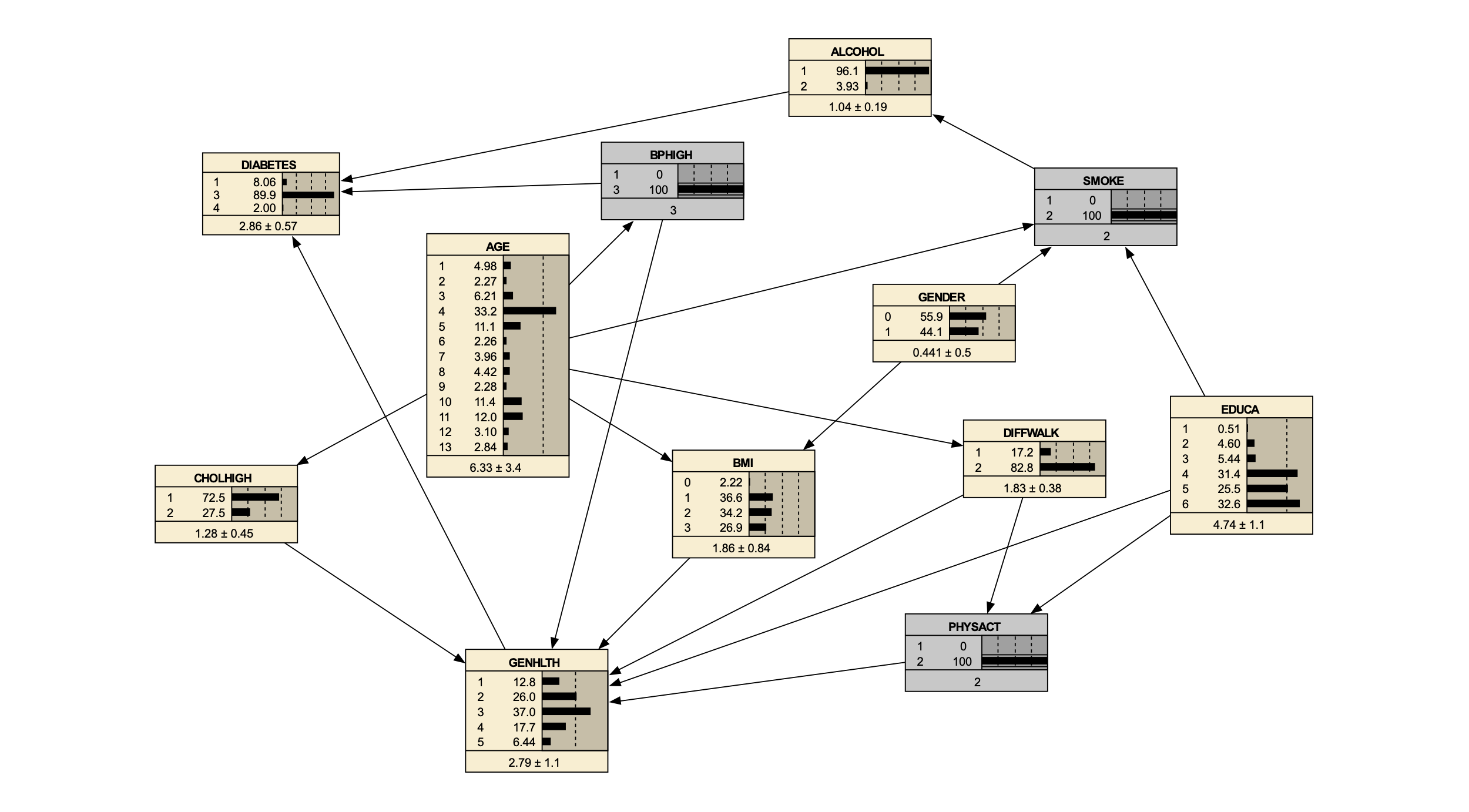


Scenario #2 Diabetes – column D


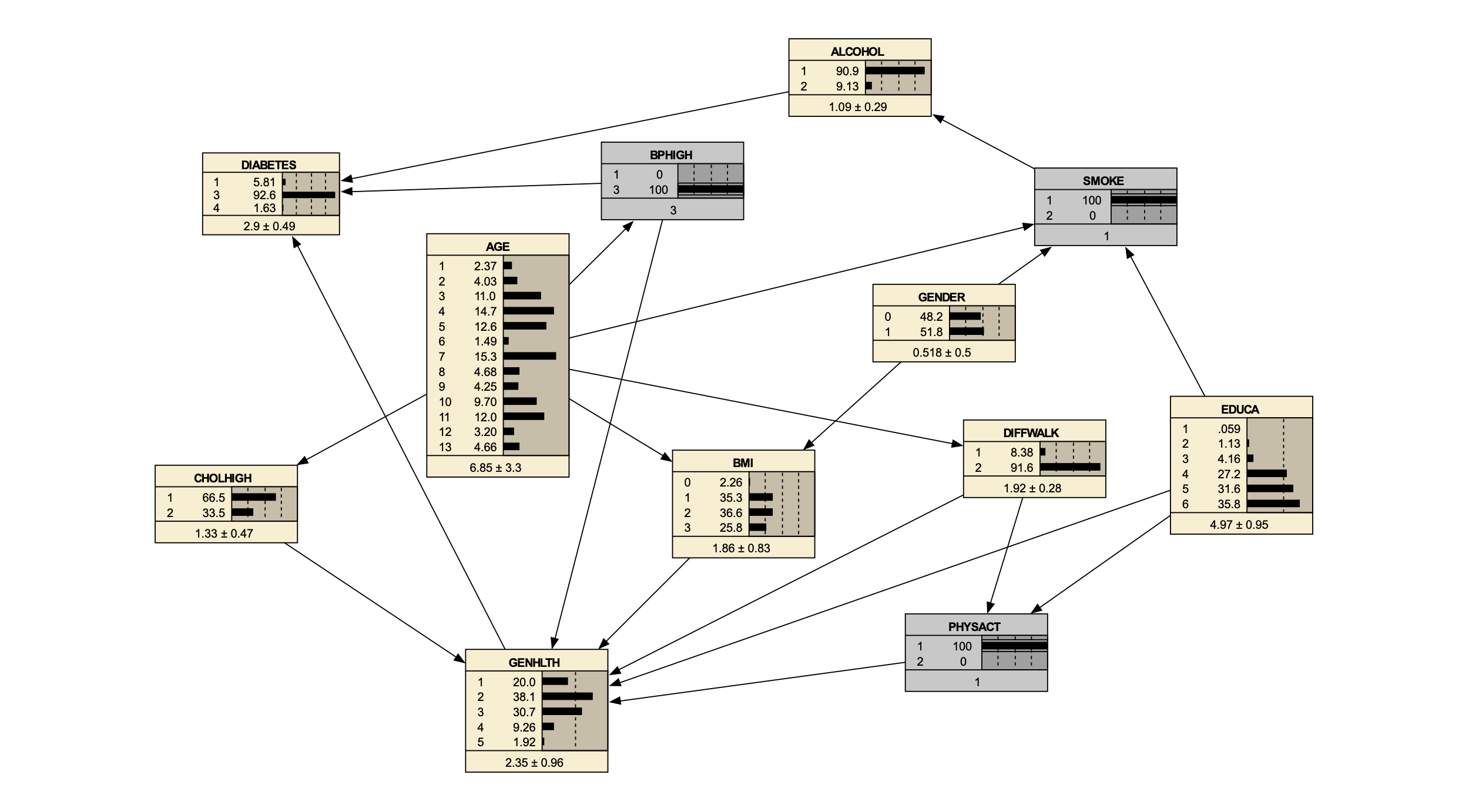


Scenario #2 Diabetes – column E


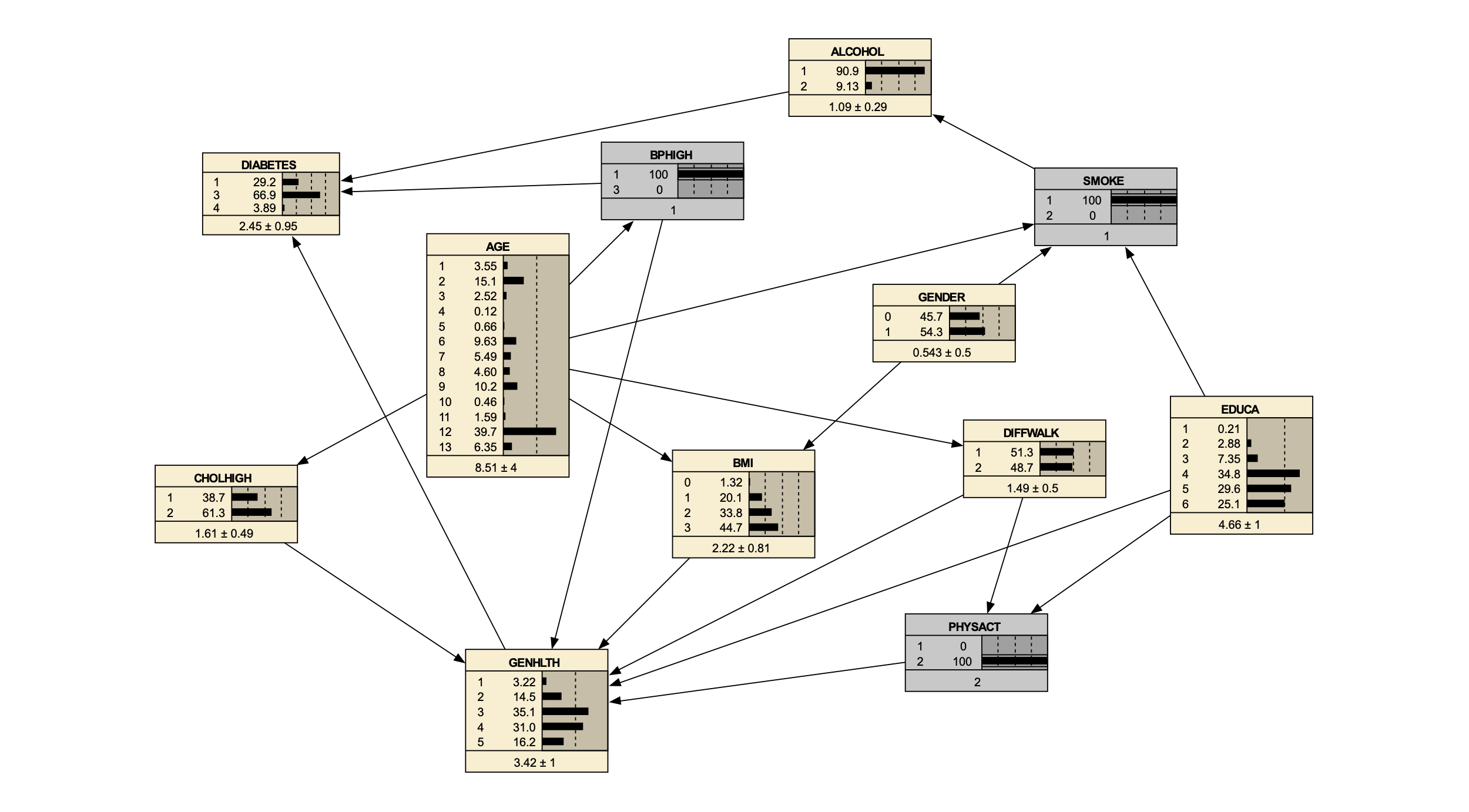


Scenario #2 Diabetes – column G


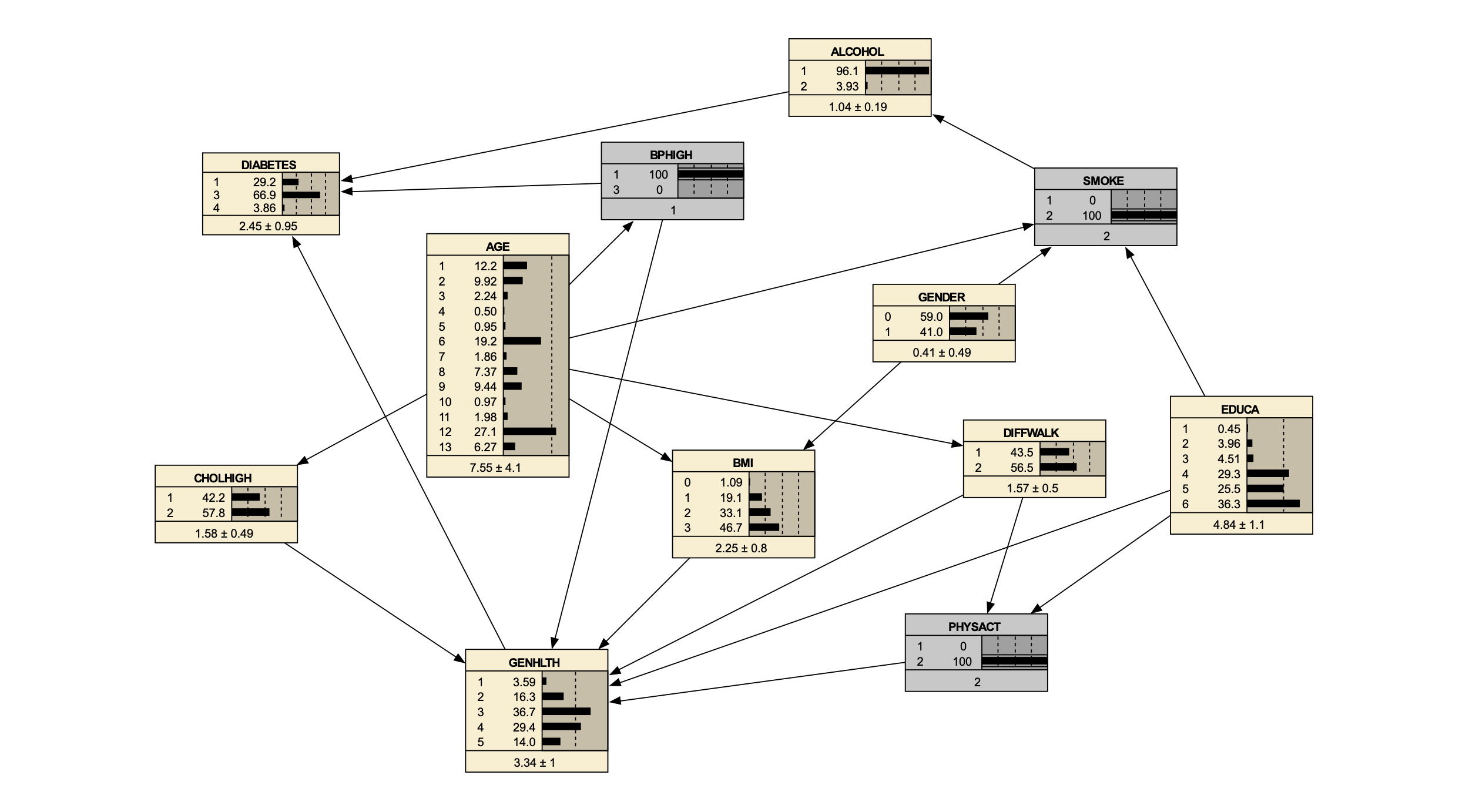


# References

1. Scutari, M. (2010). *Learning Bayesian Networks with the bnlearn R Package*. Journal of Statistical Software, 35(3), 1–22. <https://doi.org/10.18637/jss.v035.i03>
2. Norsys Software Corp. *Netica Bayesian Network Software*. Vancouver, Canada. Available: <https://www.norsys.com/netica.html>. [Accessed: 8.06.2025].
3. U.S. Centers for Disease Control and Prevention (CDC). *Behavioral Risk Factor Surveillance System: 2023 BRFSS Data*. Available: <https://www.cdc.gov/brfss/annual_data/annual_2023.html>. [Accessed: 6.06.2025].
4. Ismail, L., Materwala, H., & Al Kaabi, J. (2021). Association of risk factors with type 2 diabetes: A systematic review. Computational and structural biotechnology journal, 19, 1759-1785. <https://doi.org/10.1016/j.csbj.2021.03.003>
5. Mirzamohamadi, S., HajiAbbasi, M. N., Roshandel, G., Ghorbani, S., Badrkhahan, S. Z., Makhtoumi, M., & Zahedi, M. (2025). Incidence and predictors of type 2 diabetes mellitus during 17 years of follow-up in the Golestan Cohort Study. Scientific Reports, 15(1), 11174. https://doi.org/10.1038/s41598-025-95442-8
